# Multifunctional Nanomaterial-Integrated Hydrogels for Sustained Drug Delivery: From Synthesis and Characterization to Biomedical Application

**DOI:** 10.3390/gels11110892

**Published:** 2025-11-05

**Authors:** Magdalena Stevanović, Maja Jović, Nenad Filipović, Sara Lukač, Nina Tomić, Lana Popović Maneski, Zoran Stojanović

**Affiliations:** Group for Biomedical Engineering and Nanobiotechnology, Institute of Technical Sciences of SASA, Kneza Mihaila 35/IV, 11000 Belgrade, Serbia

**Keywords:** hydrogels, nanomaterials, drug delivery, biomedical application, material synthesis, characterization, biological interaction, data-driven strategies, responsive systems

## Abstract

Hydrogels have emerged as versatile platforms for controlled and sustained drug delivery due to their high biocompatibility, tunable mechanical properties, and ability to mimic the natural extracellular matrix. Incorporating functional nanomaterials into hydrogel networks introduces additional structural and functional complexity, enabling stimuli-responsive release, enhanced bioactivity, and synergistic therapeutic effects. This review provides a comprehensive overview of recent advances in the design, fabrication, and characterization of nanomaterial-integrated hydrogels for biomedical applications. Emphasis is placed on nanoparticle synthesis, functionalization strategies, incorporation into hydrogel matrices, physicochemical characterization, and biological aspects, including cytotoxicity, genotoxicity, antioxidative, and antibacterial effects. Emerging approaches for performance optimization, such as preliminary data-driven strategies and machine learning-based modeling, are also discussed. Special attention is given to smart and stimuli-responsive hydrogels and their potential biomedical applications.

## 1. Introduction

### 1.1. Importance of Controlled/Sustained Drug Delivery

Modern drug delivery has evolved into an interdisciplinary field at the interface of chemistry, materials science, and medicine [1]. Conventional formulations often suffer from rapid clearance, poor bioavailability, off-target effects, and frequent dosing requirements [2]. These drawbacks underscore the need for advanced delivery systems that maintain therapeutic concentrations, minimise side effects, and enhance patient compliance. Controlled and sustained drug delivery platforms address these limitations by enabling predictable, site-specific, and long-lasting drug release. Controlled drug delivery occurs when a polymeric material is combined with a drug in such a way that the release of the drug is in a predefined manner [1,3]. By maintaining drug concentrations within the therapeutic window, these systems reduce dose-related toxicity, improve pharmacological efficacy, and enhance adherence, particularly for chronic diseases [4,5]. Moreover, such systems allow the delivery of unstable [6] or narrow-therapeutic-index drugs, thereby broadening therapeutic possibilities [7,8]. Overall, controlled delivery strategies not only improve patient outcomes but also extend the clinical utility and market lifespan of pharmaceutical products [9].

### 1.2. Hydrogels as Key Platforms for Biomedical Applications

Hydrogels, three-dimensional, water-swollen polymer networks, have become essential in biomedical research due to their structural similarity to native tissues, high biocompatibility, and tunable physicochemical properties [10]. They serve as scaffolds in tissue engineering, carriers for drug and gene delivery, wound dressings, and platforms for biosensing, diagnostics, 3D bioprinting, etc. Their hydrophilic networks can encapsulate drugs, biomolecules, or cells, providing a hydrated microenvironment that promotes cell proliferation, differentiation, and tissue regeneration [11]. For example, injectable hydrogels can release anticancer drugs directly at tumor sites [12]. Hydrogels’ high-water content keeps wounds moist, accelerates healing, and reduces infection risk. Many hydrogel dressings are now loaded with antimicrobials, growth factors, or stem cells to enhance tissue repair further [13]. Hydrogels can encapsulate stem cells and deliver them to damaged tissues while protecting them from immune rejection. They can also be engineered to degrade at controlled rates, releasing cells as the scaffold gradually disappears [14]. Also, they can be engineered to respond to biological signals and changes in their environment. This makes them valuable in biosensors for glucose monitoring, disease biomarkers, and point-of-care diagnostic tools [15].

As “bioinks”, hydrogels provide the structural and biological foundation for 3D-printed tissues and organs. Their flexibility and biocompatibility make them the go-to material in additive manufacturing for biomedical research [16]. Hydrogel systems can be designed to adapt to individual patient needs, whether tailoring drug release rates, customising scaffold structures, or delivering patient-specific cell therapies. This positions them at the forefront of personalised healthcare [17]. By bridging materials science and medicine, hydrogels provide adaptable, patient-centered solutions with transformative clinical potential.

Despite these advantages, traditional hydrogels are often limited by weak mechanical strength, poor biofunctionality, and insufficient responsiveness to biological stimuli. These limitations restrict their long-term stability, control over release kinetics, and applicability in load-bearing or dynamic biological environments.

### 1.3. Nanomaterial–Hydrogel Composites: Overcoming Limitations

**Figure 1:**
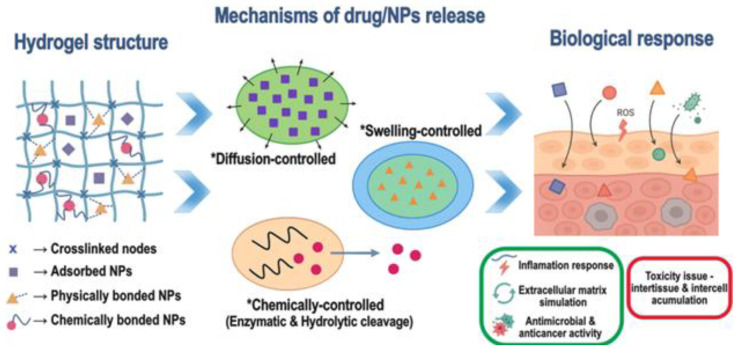
Illustration of a hydrogel incorporating nanoparticles, mechanisms of drug release, and biological response. The asterisk (*) indicates emphasis only and has no additional meaning.

Integrating nanomaterials into hydrogel matrices effectively overcomes the inherent limitations of conventional hydrogels, such as fragility, limited functionality, and inconsistent drug release, while expanding their potential in drug delivery, tissue engineering, and diagnostics. Nanoparticles (NPs) reinforce the polymer network, enhancing elasticity, toughness, and load-bearing capacity, which is crucial for applications like cartilage and bone regeneration [18]. Acting as nanoscale reservoirs or responsive carriers, NPs enable precise, controllable, and often externally triggered drug release, i.e., magnetic NPs respond to magnetic fields, while gold NPs enable light-activated delivery [19,20]. Figure 1 provides a schematic illustration of a hydrogel incorporating nanoparticles, mechanisms of drug release, and biological response.

Additionally, pH-, thermo-, and electro-responsive nanomaterials impart smart functionality for applications in tumor therapy and neural or cardiac tissue engineering [21]. Nanofiber-based systems generally offer superior mechanical stability and easier handling, making them suitable for implantable or wound dressing applications. For example, NIR-responsive shape-memory composite nanofibers can serve as deployable matrices for biomedical use, enabling spatiotemporal control of drug release upon external stimulation [22]. Beyond mechanical and responsive improvements, nanomaterials also mimic extracellular matrix components and provide bioactive cues that regulate cell behavior—hydroxyapatite NPs promote osteogenesis, while carbon-based nanomaterials support neural regeneration [23]. They further enhance imaging, sensing, and theranostic performance, while enabling fine-tuning of hydrogel porosity and degradation to synchronize drug or cell release with tissue repair [24,25]. Numerous studies have demonstrated the versatility of NPs–hydrogel systems across biomedical fields. In drug delivery, gold or magnetic NPs allow externally controlled release for cancer therapy [26], silver NPs provide sustained antimicrobial activity in wound healing [27], and nanoclay-reinforced hydrogels ensure prolonged growth factor delivery for diabetic bone repair [28]. Bioactive nanofillers such as hydroxyapatite, nanosilicates, and magnesium NPs promote osteogenesis and vascularization [29], while nanosilicate–silk fibroin hydrogels enhance chondrogenesis and bone regeneration [30]. Conductive, self-healing gold NPs-crosslinked hydrogels show promise in neural repair, including Parkinson’s and spinal cord injury models [31].

In wound management, nanomaterials confer antimicrobial, anti-inflammatory, and angiogenic effects, for example, silver, zinc oxide, and selenium NPs prevent infection and support tissue repair [32,33], black phosphorus nanosheets enable antibacterial photothermal therapy [34], and immunomodulatory NPs regulate macrophage polarization, reducing scarring and promoting regeneration [35].

**Figure 2:**
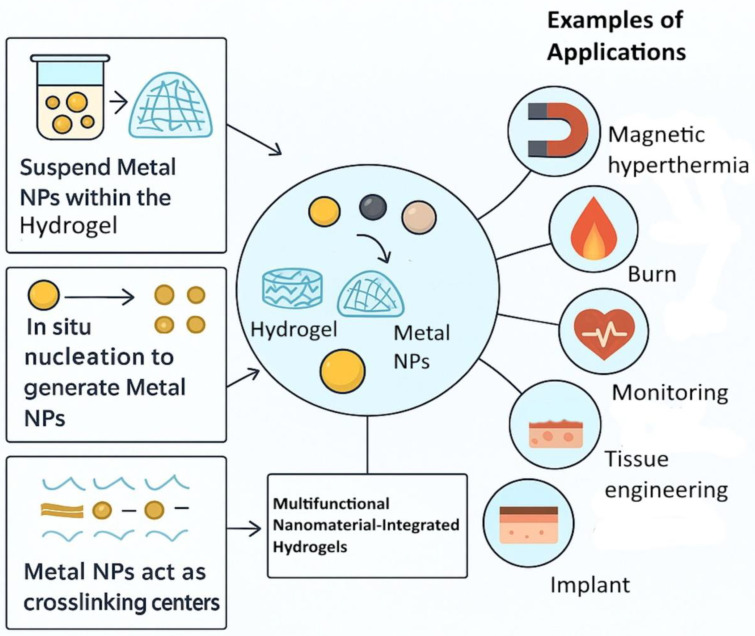
Examples of potential applications.

NPs add imaging, sensing, and signal amplification properties to hydrogels [36,37]. Figure 2 illustrates some examples. Iron oxide and quantum dots within hydrogels enable theranostic systems that track therapeutic progress [38]. Smart hydrogel dressings with color-changing probes integrated with NPs visually indicate infection or healing progress, which is important in the context of on-demand monitoring [39]. Manganese nanoparticle-loaded hydrogels provide both drug release and MRI visibility in hepatocellular carcinoma therapy [40].

Finally, in biofabrication, nanoparticle-reinforced hydrogels serve as advanced bioinks, combining mechanical stability with biological functionality. Such systems enable the creation of personalized scaffolds with tunable degradation rates and drug release profiles tailored to individual healing needs [41,42].

### 1.4. Biomedical Relevance, Research Landscape, and Methodology of this Review

Nanomaterial–hydrogel composites are not incremental improvements; they are transformative platforms that unify delivery, diagnostics, and regeneration. Their capacity to combine structural support, controlled release, responsiveness, and bioactivity positions them at the core of next-generation biomedical technologies. Whether in targeted cancer therapy, regenerative medicine, wound care, or precision diagnostics, these hybrid systems are setting the stage for more adaptive, effective, and patient-centred healthcare. These multifunctional nanomaterial-integrated hydrogels for sustained drug delivery play a crucial role in shaping the future of biomedical science, where advanced materials and nanotechnology converge to enable more precise, effective, and patient-centred therapies, as evidenced in many articles. Since 2021, approximately 16,700 research and review articles related to nanomaterial hydrogel composites have been published according to Google Scholar. This is further supported by data from the Scopus database, which shows 13,844 articles published between 2021 and 2026 under the keywords ‘nanomaterial + hydrogel + composites,’ with a publication peak in 2024, primarily in the field of materials science. These figures clearly highlight the relevance and growing significance of this research area.

The methodology for this review on multifunctional nanomaterial-integrated hydrogels for biomedical applications involves a systematic survey of peer-reviewed literature from major databases, including PubMed, Scopus, and Web of Science. Search terms combined keywords related to “nanomaterials,” “hydrogels,” “drug delivery,” “synthesis,” “surface functionalization,” “physicochemical characterisation,” “biological interactions”, “safety assessment”, “smart and stimuli-responsive hydrogels”, “data-driven design and optimization,” and “biomedical applications.” Studies were selected based on relevance to hydrogel design, integration of nanomaterials, and demonstrated or potential applications in controlled or sustained therapeutic delivery. Both experimental and review articles were considered, with emphasis on works published in the last five years to capture recent advances. The selected literature was critically analyzed and organized into thematic sections covering synthesis strategies of nanomaterials for hydrogel integration, design and fabrication of nanocomposite hydrogels, characterization and physicochemical characterization techniques, biological interactions and safety assessment, smart and stimuli-responsive hydrogels, as well as data-driven design and optimization. This approach ensures a comprehensive and structured overview while highlighting knowledge gaps and future opportunities in the field. In addition, given the growing attention this topic has received and the surge of publications over the past 5–6 years, the primary focus has been on nanocomposite hydrogels incorporating metals, metalloids, and their oxides.

## 2. Synthesis of Nanomaterials for Hydrogel Integration

### 2.1. Nanoparticle Fabrication Techniques

**Figure 3:**
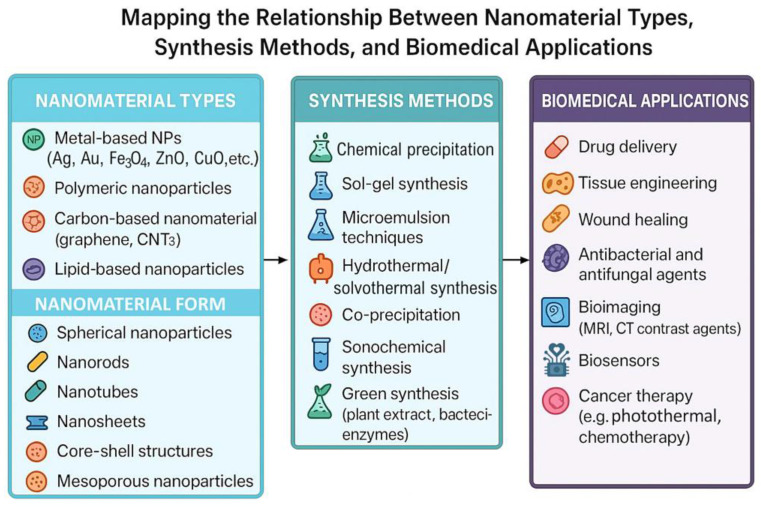
Mapping the relationship between nanomaterial types, synthesis methods, and biomedical applications.

Metal and metalloid NPs, such as silver (Ag) [43,44,45], gold (Au) [46], zinc (Zn) [47,48], chromium (Cr) [49], selenium (Se) [50,51,52], and iron (Fe) [53,54], as well as their corresponding oxides, have gained significant attention as highly valuable materials in the field of biomedicine. These nanoscale materials exhibit a wide range of functional characteristics, such as strong antimicrobial activity [55], magnetic responsiveness, and favourable surface modification potential. Their distinctive physicochemical properties, combined with the ability to be effectively incorporated into hydrogel systems, are of particular importance for various biomedical uses. Consequently, considerable effort has been devoted to developing efficient and reliable synthesis methods for metal and metalloid NPs, as well as their oxides, to optimize their properties for the specific biomedical applications. Generally, NPs synthesis techniques fall under two main strategic approaches: top-down and bottom-up. Top-down techniques involve the breakdown of bulk materials into nanoscale particles using methods such as thermal decomposition, mechanical milling, lithography, laser ablation, and sputtering [56]. The main disadvantage of the top-down approach is the imperfection of the surface structure. In contrast, bottom-up methods assemble NPs from molecular precursors, offering superior control over particle size, morphology, and surface characteristics. Common bottom-up techniques include chemical precipitation, sol–gel processes, microemulsion procedures, hydrothermal methods, co-precipitation, sono-chemical synthesis and green or biological synthesis using plant extracts or microorganisms [57] (Figure 3).

**Chemical precipitation** is the most common method for the synthesis of NPs of metals, metalloids, and their oxides. This is a very simple method of synthesis where NPs are obtained by precipitation from homogeneous precursor solutions. By adjusting synthesis parameters such as pH, temperature, ionic strength of the solution, etc., it is possible to precisely control morphology, crystallinity, particle size distribution, and surface characteristics of the obtained NPs [58]. In order to achieve stability of the obtained NPs and prevent undesirable agglomeration during or after synthesis, various surfactants are often added. The main advantages of the chemical precipitation method include its simplicity, relatively low cost, mild synthesis conditions, and the ability to influence morphology and crystallinity by carefully adjusting the synthesis parameters. These advantages make chemical precipitation a widely used synthesis method for the synthesis of NPs of metals, metalloids, and their oxides for biomedical applications. One of the recent examples is the synthesis of Ag/ZnO nanoparticles by the chemical precipitation method, followed by their incorporation into polyvinyl alcohol/pectin bioactive hydrogels for use as wound dressings [59].

**Sol–gel synthesis** is a wet chemical approach that is often used to obtain metal and metalloid NPs and their oxides. Standard sol–gel synthesis involves the formation of a sol (a solution of colloidal particles in a suitable solvent), which then develops into a gel (a solid interconnected network with submicron pores and polymer chains in the liquid phase) [60]. By drying the gel at low temperatures (up to 100 °C) and atmospheric pressure, a xerogel (porous solid matrix) is obtained. The properties of synthesized powders are affected by reaction parameters such as temperature, time, pH of the solution, type of precursor, concentration, and viscosity of the solvent. Water is the most used solvent in sol–gel synthesis, and organic solvents can also be used. With sol–gel synthesis, it is possible to precisely control the stoichiometry of the obtained NPs, morphology, particle size, degree of crystallinity and product homogeneity, which makes it suitable for biomedical applications [61]. For instance, the sol–gel synthesis of gold nanoparticles provides a versatile and effective method for precise control over particle size and distribution. A recent review by Gul et al. summarizes various Au-based nanomaterials prepared via the sol–gel approach in different morphological forms, such as spherical particles for drug delivery and star-shaped structures for diagnostic applications [46]. The drawback of sol–gel synthesis is solvent removal and gel residue elimination, which require further steps such as drying and thermal treatment, which must be carefully controlled to preserve the desired nanostructural properties.

**The microemulsion method** is a sophisticated chemical technique for synthesizing NPs with precise control over particle size, morphology, and particle size distribution. There are numerous studies on metal nanoparticles synthesized using this method. In a recent review, Dey and coauthors describe the synthesis of ZnO nanoparticles via a microemulsion system, in which water and oil phases are stabilized by surfactants to form nanodroplets that serve as nanoreactors [62]. In general, microemulsions are thermodynamically stable mixtures consisting of two immiscible liquids, one of which is usually a polar solvent, like water, and the other is a hydrophobic solvent, more commonly called oil [63]. The water–oil dispersion is stabilized by surfactants and co-surfactants, forming nanodroplets (reverse micelles) that act as nanoreactors for NPs synthesis [64]. Particle size can be precisely controlled by adjusting the concentration of surfactants, the type of oil phase, or the water/oil ratio [53]. This makes the microemulsion synthesis method highly valuable for the synthesis of monodisperse NPs of metal, metalloid, and their oxides which are ideal for applications in drug delivery, diagnostics, antimicrobial materials, and photothermal therapy. However, challenges include the use of organic solvents and complex surfactant systems, which may complicate subsequent purification and biocompatibility of the final products [65].

**Figure 4:**
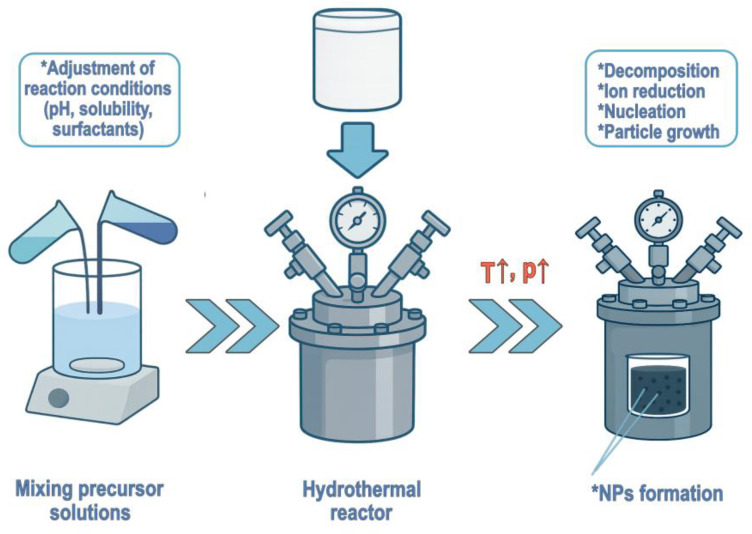
Scheme of hydrothermal synthesis. The asterisk (*) indicates emphasis only and has no additional meaning.

**Hydrothermal synthesis** is a chemical process that takes place in an aqueous solution of precursor mixtures at temperatures above the boiling point of water (Figure 4). This method is typically carried out in a closed system, an autoclave, which makes it significantly less harmful to the environment compared to other technologies for NPs production. The first step in hydrothermal synthesis usually involves mixing aqueous precursor solutions in a stoichiometric ratio. Once a homogeneous mixture is obtained, the solution is processed in an autoclave, typically at temperatures ranging from 120 to 220 °C for a specified duration. During hydrothermal synthesis, the elevated temperature of the water promotes particle diffusion, resulting in relatively rapid crystal growth. NPs of metals, metalloids, and/or their oxides can be obtained by drying the final product after synthesis.

Mineralizers may be added to precursor solutions to regulate the pH, while additives, organic or inorganic substances, at low concentrations can promote dispersion or influence the morphology of the NPs [66]. The particle size and crystal structure of hydrothermally synthesized NPs are strongly affected by synthesis parameters. The type and concentration of reactants determine the structure of the resulting particles. Higher temperatures accelerate the reaction but also tend to produce larger particles. A rapid heating rate generates numerous nucleation centers within a short time, whereas slower heat distribution allows gradual nucleation, leading to a wider particle size distribution. Thus, depending on the kinetics of particle formation, the heating rate can become a limiting factor. The pH value plays a key role, as it changes the profile of electrostatic forces on particle surfaces. Therefore, mineralizers are often required, especially when synthesizing complex nanostructures. Reaction time must be carefully optimized: insufficient duration leads to low yields, whereas excessive duration can result in the formation of larger aggregates. Pressure has an indirect effect, as it influences parameters such as solubility and density, so maintaining stable reactor pressure is essential [67]. By varying hydrothermal synthesis parameters, it is possible to obtain different morphologies of the final products. Overall, hydrothermal synthesis is a simple, clean, and relatively low-cost method capable of producing NPs of high uniformity and purity. Using the hydrothermal synthesis approach, Bîrcă et al. recently produced ZnO and CuO nanoparticles, which were subsequently incorporated into poly(lactide-co-glycolide) microspheres and embedded within collagen hydrogels with the aim of developing innovative therapeutic tools for the management of chronic wounds [68].

### 2.2. Functionalization Strategies for Compatibility with Hydrogel Systems

Integrating NPs into hydrogel matrices often faces challenges related to NPs dispersion, stability, and interactions with the polymer network. Direct incorporation of unmodified NPs can lead to aggregation, phase separation, or reduced mechanical and functional performance of the hydrogel. To overcome these limitations, functionalization strategies are employed to improve compatibility and ensure uniform distribution within the hydrogel [69,70]. The main strategies involve polymer or ligand-based surface modification, covalent conjugation, physical encapsulation, electrostatic and hydrogen-bonding interactions, as well as stimuli-responsive functionalization. Coating NPs with hydrophilic polymers (e.g., polyethylene glycol, polyvinyl alcohol) can enhance their dispersibility in aqueous hydrogel systems [71,72]. Functional ligands, such as carboxyl, amine, or hydroxyl groups, can be introduced onto NPs surfaces to promote covalent or non-covalent interactions with hydrogel polymers [73]. Chemical linking of NPs to hydrogel polymer chains can provide stable integration and prevent leaching [74]. Techniques such as click chemistry, carbodiimide-mediated coupling, or thiol-ene reactions allow specific functional groups on NPs to form strong covalent bonds with hydrogel networks, improving mechanical stability and controlling NPs release [75]. NPs can be physically entrapped within the hydrogel during polymerization or gelation [42]. While simpler than chemical functionalization, this method relies heavily on NPs size, hydrogel mesh size, and polymer–NPs interactions to maintain dispersion and avoid aggregation [76]. Introducing charged groups onto NPs surfaces allows electrostatic attraction with oppositely charged hydrogel polymers [77]. Hydrogen bonding between functional groups on NPs and hydrogel chains can further enhance compatibility and uniform dispersion [78]. NPs can be modified to respond to pH, temperature, or light, enabling dynamic interactions with the hydrogel matrix [79]. This approach is particularly useful for controlled drug delivery, where NPs release can be tuned in response to environmental triggers.

Functionalization is essential for achieving homogeneous NPs distribution, enhanced stability, and tailored interactions within hydrogel systems. Properly functionalized NPs can significantly improve the mechanical, thermal, and bioactive properties of hydrogels, enabling applications in drug delivery, tissue engineering, and biosensing.

## 3. Design and Fabrication of Nanocomposite Hydrogels

Driven by specific application needs, researchers are continually enhancing and customizing hydrogel properties to surpass those of their individual components. As noted above, one strategy in this endeavor involves the incorporation of multiple components, particularly NPs. Numerous studies have highlighted that the design, choice of constituents, and overall synthesis process of composite hydrogels are critical factors in determining their application potential. Hydrogels can be classified depending on their composition, network structure, properties, response to external conditions, and other parameters. They can be classified by polymer source (natural, synthetic, or hybrid), composition (homopolymeric, copolymeric, or interpenetrating networks), bonding mechanism (chemically or physically crosslinked), microstructure (micro- or nanocomposites), and form (beads, films, matrices, or injectable gels). The mechanism of crosslinking strongly determines the structural, mechanical, and swelling behavior of the hydrogel. As mentioned above, two general principles are utilized in hydrogel production: **physical** and **chemical** crosslinking. **Physical crosslinking** involves non-covalent interactions, including hydrogen bonding, ionic interactions, crystallite formation, hydrophobic association, or supramolecular self-assembly. These gels are generally reversible, as the interactions can be disrupted and reformed under changing environmental conditions such as pH, temperature, or ionic strength. For example, by subjecting polymer solutions to freeze–thawing cycles during which phase separation and crystallite formation act as junction zones that stabilize the three-dimensional network, it is possible to form a stable hydrogel without using any chemical crosslinkers. In such cases, the critical gelation concentration defines the lowest polymer concentration at which a continuous network is formed. As a type of physical crosslinking approach, **ionic complexation** (polyelectrolyte interactions) is commonly used in hydrogel production. Networks are created when oppositely charged polymers or polymer ions associate to form stable complexes. The degree of crosslinking and the resulting gel strength depend strongly on polymer concentration, charge density, and ionic environment, again giving rise to a critical gelation threshold. One of the most popular examples of hydrogels used in the biomedical field that undergoes this mechanism of crosslinking is alginate/metallic bivalent cation.

**Chemical crosslinking**, in contrast, creates permanent covalent junctions between polymer chains. This can be achieved through free-radical polymerization of functional groups, condensation reactions forming amide or ester linkages, or high-energy irradiation that induces bond formation. Click chemistry approaches, such as azide–alkyne cycloaddition, also provide efficient and selective routes to network formation. A particularly important chemical pathway is the Schiff base reaction, in which aldehyde or ketone groups react with amines to form dynamic imine linkages; these bonds are reversible under physiological conditions, which makes Schiff base hydrogels attractive as self-healing and injectable systems. Unlike physical gels, chemically crosslinked hydrogels are less sensitive to external fluctuations and show improved stability and mechanical resistance, although they lack reversibility.

**Enzymatic crosslinking** represents another route that can be considered as a type of chemical crosslinking, important particularly for systems where mild conditions are required. Enzymes catalyze bond formation between functional groups on polymer chains, allowing gelation under near-physiological conditions. These reactions are highly specific and enable the construction of hydrogels without harsh chemical initiators.

Overall, hydrogel formation can be understood as the establishment of a percolating network once a sufficient number of junction points is created, whether through non-covalent, covalent, enzymatic, or ionic interactions. The concept of a critical gelation concentration is central, as it defines the transition from a viscous solution to a self-supporting solid-like network. By adjusting crosslinking mechanisms and conditions, a wide spectrum of hydrogel structures can be obtained, ranging from soft and reversible to highly stable and mechanically robust.

The mechanisms of crosslinking and routes for hydrogel production are a pervasive area that itself could be the topic of a separate review. Numerous papers that extensively cover aspects of this topic can be found elsewhere [79,80,81,82,83,84,85].

### 3.1. Incorporation of Metalloid, Metal, or Metalloid/Metal Oxide Nanoparticles

To date, several strategies for incorporating metal and metal oxide NPs into hydrogels have been developed, each with its own strengths and limitations. The choice of method depends on the desired properties of the final material, such as mechanical stability, drug release behavior, or responsiveness to external stimuli.

One of the commonly used approaches is in situ synthesis, where metal ions are introduced into the hydrogel network and then reduced directly within the gel. This method allows NPs to form inside the three-dimensional polymer matrix, resulting in excellent dispersion and strong interactions between the particles and the polymer chains. Because the NPs are produced within the gel, their size and prevention from agglomeration can be controlled. However, the reaction conditions must be carefully optimized to prevent damage to the hydrogel structure. In the work of Zhenhu Guo et al. [86], the authors reported the production of a composite hydrogel in a fast and eco-friendly method. In this work, Fe^3+^ ions were selected as inorganic cross-linkers to link gelatin and protocatechuic acid into gelatin–metal–polyphenol hydrogel. Thanks to the reducing properties of phenolic groups, Fe^3+^ ions were in situ effectively transformed into NPs [86]. Upon near-infrared (NIR) irradiation, this composite hydrogel was capable of transforming light into heat energy for inducing tumor cell apoptosis. The influence of numerous parameters on in situ formation of NPs was thoroughly investigated in the work of Gail A. Vinnacombe-Willson and colleagues [87]. They introduce a novel approach for synthesizing gold nanostars directly on gelatin-based hydrogels through an in situ seed-mediated growth strategy, avoiding conventional colloidal synthesis or self-assembly routes. By systematically varying polymer composition, gold precursor concentration, and seeding conditions, the authors demonstrated precise control over nanoparticle morphology and optical properties, tuning them into the NIR range. The study also explored the role of functional groups and crosslinking mechanisms in supporting or hindering in situ growth, highlighting both challenges, such as secondary nucleation, and practical solutions like optimized curing and washing steps. Importantly, the method enabled surfactant-free synthesis in biocompatible media (PBS), yielding nanostructures suitable for cell culture and biosensing applications as well. Overall, in situ growth of NPs is a commonly used technique for the preparation of versatile composite hydrogels [88,89,90]. The more straightforward technique is to **mix pre-synthesised NPs** with the polymer solution before gelation. In this approach, NPs are added to the polymer solution, and the mixture is then crosslinked to form the hydrogel, effectively “trapping” the particles inside. This method is straightforward and works well with sensitive biomaterials, but the interactions between the particles and the matrix are usually weaker [91,92]. Stabilizing agents are sometimes needed to prevent aggregation during gel formation.

In contrast with the previous method, **post-gel functionalization** offers more flexibility. Here, the hydrogel is first formed and then mixed with NPs, leading to covalent bonding, electrostatic interactions, or simple adsorption [93,94,95,96]. This allows researchers to fine-tune the hydrogel surface and add functionality after its structure is already established. The possible drawback of this approach is the lack of homogeneous distribution of NPs within the entire hydrogel volume and/or random aggregation of NPs into the polymer matrix.

More advanced materials can be created through **self-assembly methods**, where NPs and polymers organize themselves into structured networks driven by noncovalent/covalent interactions. For example, the Schiff-base reaction between chitosan and 4-formyl benzoic acid was used for the production of a self-assembled hydrogel with incorporated AgNPs through in situ condensation and reduction [97]. As the authors reported, the gelation process was enhanced by the crosslinking of AgNPs within the hydrogel network. This hydrogel was designed to be capable of pH-responsive delivery and release of doxorubicin and paclitaxel. Due to the supramolecular organization, the self-assembled hydrogels are especially interesting from the aspect of reversible bonding and ability to self-heal or respond to environmental changes [98]. Another interesting example can be found in the paper published by Weihong Chen et al. [99], where peptide precursor Nap–Phe–Phe–Tyr was used for spontaneous self-assembly through π–π stacking and hydrogen bonding. This fibrous supramolecular network was further used for in situ precipitation of Au NPs, yielding a composite with a uniform distribution of NPs. The ordered architecture enhanced both the antibacterial performance and the wound-healing efficiency compared with the hydrogel or Au NPs alone, demonstrating that the cooperative self-assembly produced a synergistic improvement in stability and therapeutic function.

Another strategy involves **polymerizing monomers** in the presence of NPs, allowing the NPs to act as anchoring points or nucleation sites during gel formation [100,101]. This approach creates a strong connection between the NPs and the hydrogel matrix, which enhances stability and durability. However, it also requires careful consideration of polymerization chemistry, as initiators or reaction conditions can sometimes harm biological components or reduce biocompatibility. To avoid this issue, a potential approach could be the utilization of sunlight and NPs [102]. As reported in this paper, acrylamide was used simultaneously as a reducing/capping agent to form AuNPs and as the monomer that polymerizes into the hydrogel network. The polymerization was initiated via localized surface plasmon resonance of in situ-formed AuNPs. This specific internal organization enables the formation of a nanocomposite hydrogel under relatively low monomer concentrations, while maintaining a well-defined porous structure. Similarly, the improvements of mechanical resilience under compression and elongation, self-healing, and an ability to recover shape rapidly under temperature and light stimuli as a result of internal organization were reported recently [103]. According to the authors, nanocomposite gels can be prepared via bulk free-radical polymerization of stearyl methacrylate and vinyl pyrrolidone in the presence of AuNPs or AgNPs, without using chemical crosslinkers. The stearyl methacrylate provides hydrophobic long alkyl side chains, vinyl pyrrolidone contributes dipole–dipole interactions, and together with the NPs incorporated before curing, they form a physically crosslinked network.

Finally, **layer-by-layer (LbL) assembly** and multi-network hydrogels offer a way to design complex materials with high mechanical strength and multiple functionalities. By forming hydrogels in several layers or creating multiple interpenetrating networks, researchers can incorporate NPs at different stages of gel construction. For instance, Kun Yan and colleagues reported a layer-by-layer electrochemical approach to fabricate chitosan/silver NPs multilayer hydrogels [104]. In their work, chitosan was electrodeposited on a stainless-steel electrode under a pulsed electrical signal, producing an organized multilayer architecture. Silver ions were then adsorbed into the hydrogel and reduced in situ by electrochemical polarization, yielding uniformly distributed ~15 nm AgNPs stabilized within the polymer matrix.

The resulting nanocomposite retained its multilayer structure, which enabled pH-dependent staged release of AgNPs, while the embedded NPs imparted strong and sustained antibacterial activity. In another example, LbL was achieved via electrostatic interactions between layers of polyelectrolytes, doxorubicin, and camptothecin-loaded gold nanorods [105]. These layers were deposited onto preactivated silicon substrates and showed dual effect: pH-dependent release of doxorubicin and camptothecin release upon NIR irradiation, driven by the photothermal activity of gold nanorods. Importantly, the films exhibited excellent cytocompatibility and anticancer activity, highlighting LbL assembly as a versatile and dual-stimuli-responsive drug delivery platform. As can be concluded LbL approach opens the door to designing “smart” materials capable of controlled drug release, real-time monitoring, or multiple therapeutic actions. Although these systems can be time-consuming to prepare, they provide some of the most advanced performance characteristics available in hydrogel-based biomaterials.

One of the latest approaches in designing hydrogels that has emerged recently and can be considered as a type of LbL technique is the electrofabrication. With this additive manufacturing, by applying electrical signals, it is possible to control how polymer chains arrange and move, and more importantly, how ions spread from electrodes. Metal ions driven by the electric field can bind with the polymer, creating local crosslinking points that strengthen the hydrogel [106]. Furthermore, these metal-ion coordination sites can act as starting points for crystal growth. Metal oxides can grow directly on such sites, which opens the possibility of integrating functional metal oxides into hydrogels through in situ crystallization [107]. In addition, the type and size of the resulting metal oxide NPs can be adjusted by changing the metal electrodes and the growth conditions. This approach provides a useful pathway for producing soft robotic systems that include built-in magnetic components.

Taken together, all mentioned techniques provide a versatile toolkit for designing NPs-loaded hydrogels. Simpler methods, such as in situ synthesis and pre-gel mixing, are widely used in biomedical research because they are easy to perform and provide reliable integration of NPs. On the other hand, more sophisticated approaches like self-assembly, in situ polymerization, and multi-network systems are increasingly popular for applications where precise control, responsiveness, and multifunctionality are required. By selecting the right strategy—or even combining several—researchers can fine-tune hydrogels to act as drug delivery platforms, tissue scaffolds, biosensors, or smart therapeutic materials. The summary of basic information regarding the preparation of composite hydrogels is listed in Table 1.

### 3.2. Tunability of Mechanical, Swelling, and Release Properties

**Figure 5:**
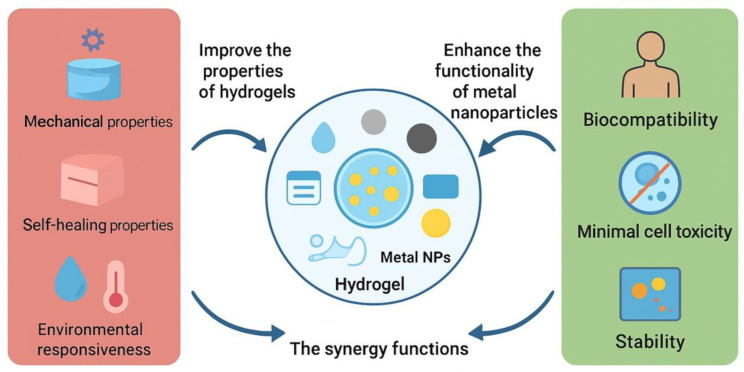
Some of the advantages of nanomaterial-integrated hydrogels.

As it was already mentioned, the incorporation of NPs into hydrogels has emerged as an effective strategy to overcome the inherent limitations of polymer networks, such as their weak mechanical strength, limited stability, and uncontrolled drug release [25,108,109] (Figure 5). Metal and metal oxide NPs can serve as physical or chemical crosslinking centres, reinforcing the hydrogel structure, improving viscoelasticity, and extending degradation resistance [110]. At the same time, their unique surface chemistry and responsiveness enable more controlled and sustained drug release, transforming conventional hydrogels into multifunctional delivery platforms. In addition, NPs–hydrogel systems often exhibit stimuli-responsive behavior, where external triggers such as light, temperature, pH, or magnetic fields can finely tune network swelling or drug release. This dynamic responsiveness further broadens their biomedical potential, allowing hydrogels to act as adaptive and intelligent materials for therapeutic applications.

Samaneh Khodami, with colleagues, reported a significant multifunctional improvement of poly(N-isopropylacrylamide) hydrogels by incorporating linker-modified gold NPs [111]. The presence of AuNPs reinforces the polymer network, leading to significantly higher tensile strength and rapid self-healing, with recovery of nearly 85% within just two minutes under NIR irradiation. At the same time, the NPs contribute to fast thermo-responsive behavior and enable precise control over drug release, achieving delivery efficiencies almost twice as high as those of NPs-free hydrogels. Another notable feature is the strong adhesion of these composite gels to skin-like substrates, which proves their potential for applications in wound dressings and wearable therapeutics. Altogether, the integration of AuNPs provides a synergistic effect (enhancing mechanics, adhesion, self-repair, and controlled release), making these hydrogels highly attractive for next-generation biomedical use.

When it comes to improvements of mechanical properties thorough study that addressed this issue was done by Alvaro Charlet et al. [112]. In their work, telechelic PEG hydrogels functionalized with pyrogallol end groups (2gPEG) were crosslinked with several di- and trivalent metal ions, and the crosslinking mechanism as well as influence on mechanical properties was investigated. Upon raising the pH, the pyrogallol groups deprotonate and interact with metal ions, leading to precipitation into NPs that serve as crosslinking sites. This mechanism increases the effective crosslink density, giving the hydrogels solid-like viscoelastic behavior and allowing the formation of freestanding 3D structures with load-bearing capacity, even underwater. Trivalent ions such as Fe^3+^, Al^3+^, and V^3+^ yield hydrogels with comparable stiffness, while divalent ions show a much wider range of responses, with mechanical strength increasing with the electronegativity of the ion (from Ca^2+^ to Cu^2+^). The process is reversible: lowering the pH disrupts crosslinking and liquefies the gel, while Fe_3_O_4_ NPs can be introduced to further extend functionality, such as magnetic responsiveness. Similarly, Yi Li and colleagues have shown that in situ generation of silver NPs within dopamine-conjugated gelatin significantly improved its performance compared to nanoparticle-free controls [113]. Hydrogels containing Ag NPs exhibited higher stiffness and toughness, showing greater resistance to deformation while maintaining flexibility. Their adhesion strength under wet conditions was markedly superior, since the NPs served as multifunctional crosslinking points that stabilized the interface. Moreover, while plain hydrogels showed only short-term antibacterial activity, those with Ag NPs provided a more sustained release profile, ensuring prolonged protection and therapeutic effectiveness.

The use of transition metal oxides (TMO) such as iron oxide (Fe_3_O_4_), titania (TiO_2_), zirconia (ZrO_2_), etc., in the biomedical field has found its way in the hydrogel design, as well. There is a comprehensive review paper regarding this topic that highlights the benefits of TMO hydrogels for the treatment of diabetic bone defects and wound healing [114]. As stated in this review, the design of TMO-decorated hydrogels can improve controlled delivery as a response to particular stimuli typical for diabetic wound conditions, such as temperature, pH, or enzyme activity changes. Additionally, the incorporation of TMOs within hydrogels increases mechanical properties, bioactivity, and cell proliferation. For instance, in the paper published by Shikha Awasthi et al. [115] it was demonstrated the synergistic effect and strong interfacial bonding of TiO_2_ NPs and carbon nanotubes to the polyacrylamide (PAM) hydrogel. This nanocomposite hydrogel was prepared by mixing the dried PAM hydrogel with nanoparticle suspensions, which led to the formation of H-bonding and Van der Waals-type interactions, and finally resulted in mechanical properties improvement- higher compressive strength, elastic modulus, and puncture resistance. As PAM−TiO_2_−CNT composite hydrogel expressed high self-healing (recovered insertion depth up to 25 μm), without compromising the bioactivity, and cytocompatibility, it could be considered for cartilage repair applications. Another example of successful utilization of TMO is to enhance drug release upon external stimuli. The combination of Fe_3_O_4_ NPs and hydrogel in the so-called “ferrogels” provides the magnetic property of these composite gels. As Shabnam Nezami et al. presented in their work, this approach allowed them to achieve increased release of Guaifenesin from 54.1% up to 90.4% by applying an external magnetic field [116]. Similarly, by integrating Fe_3_O_4_ NPs, G.R. Bardajee et al. achieved enhanced mechanical stability of a multicomponent hydrogel (poly(N-isopropylacrylamide-co-acrylamide)-poly(acrylic acid)), and tunable release of Doxorubicin through external magnetic fields [117]. In another work, Amin Karimi-Rastehkenari et al. [118] reported the increase in swelling rate for Chitosan/Hyaluronic acid hydrogel thanks to the incorporation of Fe_3_O_4_ NPs. As a result, the antibiotic drug Ciprofloxacin was entrapped with 93% efficiency and released 100% within 13 h [118]. More details regarding the application of ferrogels in biomedicine can be found somewhere else [119,120].

The alteration of drug release by external stimuli was reported in another study conducted by Fenfen Zhang et al. [121]. In this work, the authors reported a specific design of composite hydrogel (bioink for 3D printing) based on gelatin methacrylate with the addition of deferoxamine-loaded Au NPs and N-isopropylacrylamide. As the authors stated, the incorporation of AuNPs reinforced the hydrogel network through enhanced hydrogen bonding, ensuring sufficient mechanical strength. Additionally, when exposed to 808 nm NIR light, this multifunctional hydrogel-scaffold demonstrated mechanical contraction, which further promoted the release of DFO and AuNPs. NIR light-responsive hydrogels are a promising strategy for enhanced antimicrobial and antioxidative activity, especially used in wound healing [92,122].

The burst release of the hydrogel’s active loading is considered as a huge drawback for this type of biomaterial. One possible route to overcome this issue is employing the mesoporous silica nanoparticles (MSNs). In a recent study, MSNs were incorporated into a GelMA/PEGDA hydrogel network loaded with insulin-like growth factor 1 (IGF-1) and vancomycin, leading to notably improved structural stability and sustained multi-drug release (after 144 h, still releasing IGF-1) [123]. Overall, MSNs emerged as a promising strategy in the design of hydrogel-based drug delivery systems due to their highly tunable size, composition, surface chemistry, and porous architecture, which enable efficient loading of therapeutic agents and protection of labile drugs. Moreover, MSNs can serve as reinforcing and crosslinking points in hydrogels, increasing network density, mechanical strength, and overall structural stability. There is an excellent review paper, published recently, that highlights all these benefits of MSNs [124].

Another example demonstrating the influence of hydrogel composition and structure on drug release can be found in a paper published by Roberto Bernasconi et al. [125]. In this work, 3D-printed and metallized microdevices were successfully functionalized with multilayered hydrogel coatings using a layer-by-layer deposition of alginate and oppositely charged polyelectrolytes (e.g., chitosan or poly(allylamine) hydrochloride), crosslinked with Ca^2+^ ions. This stratified assembly not only provided stable hydrogel shells but also significantly improved drug release control by reducing burst release and prolonging release duration, due to the increased diffusion path created by multiple bilayers. Interestingly, the thicker hydrogel coatings altered the devices’ shape and actuation, requiring higher magnetic fields for motion, but they still enabled controlled movement, highlighting their potential as targeted drug-delivery microrobots.

In some cases, the utilization of NPs from hydrogels can improve the activity of the drug, embedded in a polymer matrix, as well. For instance, in the work done by Ilknur Atli et al [126]. AuNPs were incorporated within zei-agarose hydrogel along with Silibinin. This resulted in synergistic anticancer effect on colon cancer (HT-29) cell lines along with additional antimicrobial effects [126].

Driven by the advantages of the electrofabrication strategy, Hang Zhao et al. recently reported the development of protein-based hydrogel soft robots designed for active drug delivery [127]. The authors used silk-elastin-like recombinant proteins that respond to temperature changes for hydrogel production. This hydrogel was further placed between two electrodes, Fe as anode and Al as cathode. After applying an electrical field, Fe^2+^ ions were homogeneously distributed and caused the coordinative bonding within the hydrogel. By introducing the source of Fe^3+^ ions and alkaline conditions, these coordination sites were used for in situ crystallization of Fe_3_O_4_ NPs. The embedded NPs (~8 nm) both strengthen the hydrogel network and provide superparamagnetic properties, allowing remote control and guided movement through confined environments. Mechanical improvement was verified by comparing the empty hydrogel, hydrogel with electrochemically induced crystallization of Fe_3_O_4_ NPs, and hydrogel with regularly crystallized Fe_3_O_4_ NPs. Results from rheology and compression tests showed that Fe_3_O_4_–hydrogels made with electrochemical crystallization had the highest mechanical strength. This improvement is linked to stronger crosslinking from Fe–carbonyl coordination sites and the formation of additional networks during in situ Fe_3_O_4_ crystallization. Moreover, their strong photothermal response enables NIR triggered drug release. Ex vivo studies with porcine intestines confirm the potential of these magnetic soft robots for targeted navigation and controlled drug delivery.

Sometimes, hydrogel can be upgraded by embedding two types of NPs. For instance, in the study published by Xingkai Ju et al., PEG-coated ZnO nanorods and Au NPs were incorporated within a polyionic liquid-based hydrogel [128]. Due to the piezoelectric properties of ZnO, electrical stimulation was achieved under ultrasonic irradiation. Besides improved antibacterial activity, the presence of NPs increased swelling capacity, enhanced ROS generation, and overall faster wound healing, confirmed by in vivo experiments. Another example is the incorporation of CuO and ZnO NPs within a hydrogel composed of sodium alginate, polyvinyl alcohol, and gelatin. The presence of both NPs increased porosity and swelling capacity and resulting in enhanced antimicrobial properties [93]. Furthermore, an interesting paper was published recently where it was demonstrated that there was a dual benefit of Bi and CeO_2_ NPs embedded within a Schiff-base chemically crosslinked hydrogel composed of aldehyde-based hyaluronic acid and gelatin [129]. The termed ZIF-90-Bi-CeO_2_ NPs enhanced antibacterial and antibiofilm activity through photothermally induced release of Zn ions and CeO_2_ NPs, and controlled regulation of ROS levels due to the enzyme-mimicking properties of CeO_2_. The application potential of this multifunctional hydrogel was confirmed in a rat implant-associated infection model. Due to the covalent and non-covalent bonding between gelatin and polydopamine, the hydrogel adhered firmly onto the polydopamine-modified titanium implants. Beyond improving antimicrobial efficiency, the coated implants had a biofilm-removal ability and regulated oxidative stress and inflammatory responses to facilitate osteointegration.

In addition to improving drug delivery performance, the inclusion of NPs could enhance the self-healing ability of hydrogels through reversible noncovalent interactions between polymer chains and nanoparticle surfaces. Such an example can be found in the paper published by Yating Liu et al. [130]. In this study nanocomposite hydrogel was formed with hyaluronic acid and alginate crosslinked through reversible Schiff base bonds. The authors reported excellent self-healing ability, with damaged gels spontaneously restoring their structure and recovering nearly 90% of their mechanical strength. Incorporation of cuprous-benzoylthiourea (BTU)-modified Ag NPs further enhances the hydrogel’s functionality by enabling continuous ROS generation and Ag^+^ and Cu^+^ release for strong antibacterial effects. Besides stabilisation of Ag NPs and copper ion chelation, BTU promoted the cyclic conversion between Cu^2+^ and Cu^+^ and enhanced chemodynamic antibacterial therapy. Other examples of hydrogel properties improvement are summarised in Table 2.

As a key takeaway from this section, future designs of nanocomposite hydrogels should carefully consider the nature of interfacial interactions between nanoparticles and the polymer matrix, as these interactions play a crucial role in determining the structural integrity, mechanical performance, and functional properties of the final system. Through covalent bonding, coordination interactions, hydrogen bonding, or electrostatic attractions, nanoparticles can act as multifunctional crosslinking nodes that enhance the network density and improve the load transfer between the inorganic and polymeric phases. These interactions significantly reinforce the hydrogel’s mechanical strength, elasticity, and resilience, often leading to higher storage moduli and improved resistance to deformation. Additionally, well-designed interfaces can modulate water uptake and swelling behaviour by influencing the hydrogel’s internal architecture, while also stabilising the network and reducing premature degradation. Beyond structural reinforcement, the nanoparticle–polymer interface directly impacts drug release profiles. For instance, controlled binding or confinement of therapeutic agents at the interface enables sustained and stimuli-responsive release, reducing burst effects and improving therapeutic precision. This synergistic interplay between nanoparticles and polymer chains is central to tailoring hydrogel properties for advanced biomedical applications.

To facilitate a clearer understanding of the concepts presented in this section, Table 3 offers a comparative summary of various nanocomposite hydrogels and outlines how specific types of nanomaterials dictate their key properties. In addition to mechanical strength, release behaviour, and responsiveness, the table also includes essential information on biocompatibility and potential toxicity, as these parameters are critical for the safe design of materials intended for biomedical use. A comprehensive discussion on this topic is provided in Section 5. This summary may also serve as a practical guide for researchers when selecting suitable nanomaterials for specific biomedical goals.

## 4. Physicochemical Characterization Techniques

Thorough characterization of hydrogels is crucial to comprehend, optimize, and utilize their properties for later applications. As no single characterization method may give a full picture of their features, this section will present an overview of some of the most common characterization methods used to evaluate the properties of hydrogels, with special emphasis on physicochemical methods, as well as considering their potential limitations.

### 4.1. Microscopy Techniques

Scanning electron microscopy (SEM) is generally reported as the most widely used method for characterizing hydrogel microarchitectures. This tool provides a detailed visualization of the hydrogel surface at the nanometer scale [149]. The morphological characterization of hydrogel-based materials is essential for monitoring their size, shape, surface texture, and internal structure [150]. SEM microphotographs of hydrogels are employed to determine pore size, pore distribution, and porosity percentage, as well as fiber thickness and fiber orientation [149]. More specifically, varieties of SEM commonly used for the evaluation of hydrogels include cryogenic (Cryo-SEM) or high-vacuum scanning electron microscopies [150].

The limitations of SEM arise during the hydrogel preparation steps, as visualization requires a dry specimen. Thus, SEM is inherently biased, as desiccation will alter the native microarchitecture of a hydrogel [149].

Compared to the SEM techniques, the transmission electron microscopy (TEM) gives details on the hydrogels’ internal structure, yielding extremely high-resolution details on material structure and morphology [151]. It may provide excellent resolution for observing the nanometric features of the hydrogels. Moreover, unlike SEM or Cryo-SEM, which primarily provide topographic information, TEM allows for deeper penetration into samples: it can, for example, reveal the internal details of materials encapsulated within hydrogels [152]. It is especially effective for inspecting the distribution of inorganic substances and release kinetics in hydrogel-filled devices, confirming their depth and distribution patterns [153].

However, it is worth noting that applying TEM to observe the network structure of hydrogels that consist of thin, flexible polymer chains swollen in water is highly challenging. Existing TEM observation methods, such as electron staining, phase contrast, and scanning TEM, provide difficulties in giving sufficient electron density contrast for individual polymer chains of synthetic hydrogels [154].

### 4.2. Spectroscopy Techniques

The Fourier-transform infrared spectroscopy (FTIR) is a common physicochemical characterization technique that finds its application in hydrogel research, whose primary aim is to determine the specific chemical groups and bonds in the structures of hydrogels and materials [155]. Therefore, it is a valuable asset in determining the synthesis mechanism for the production of hydrogels [155]. It can confirm the successful crosslinking of reactants, hydrogel network formation, and drug loading [156]. It can also be used for assessing phase transitions, via observing the changes in the hydration of carbonyl groups in polymer chains [157]. It is also worth mentioning that the FTIR technique, especially accompanied by AFM, can be used for the determination of optical properties such as refractive index [158].

The presence of water within the hydrogel matrix may cause problems in detecting the different bonds and vibrations within the polymeric chains. For that reason, analysis is usually performed on dried hydrogels [159].

Nuclear magnetic resonance (NMR) spectroscopy is a non-invasive characterization technique used to gain detailed insights into the structures of the functionalized hydrogels. It has been extensively used for in-depth structural characterization of soluble polymers, including the determination of their compositions, microstructures, and, more recently, molecular weights [160]. NMR can also be used to monitor the gelation process of supramolecular hydrogels [151].

However, hydrogels are generally insoluble, which precludes the use of many analytical methods, such as nuclear magnetic resonance (NMR) [161].

Raman spectroscopy, a technique complementary to the IR methods, can provide detailed information on chemical structure and molecular interactions, used to evaluate the composition of hydrogels at the molecular level [162]. In hydrogels, Raman spectroscopy has proven to be a valuable tool for the evaluation of polymer-water molecular interactions. In particular, the activity of the OH stretching band can be used to obtain information about the structure of imbibed water [163]. It is also used to study the extent of polymer gel formation and crosslinking within tissue [164]. Another use of Raman spectroscopy is to evaluate the presence of a specific element within the hydrogel [163].

The main disadvantage of using Raman spectroscopy lies in the fact that the Raman effect, which lies in the foundation of the technique, is inherently low, thus resulting in low signal efficiency [165].

### 4.3. Thermal Methods

Thermal properties can be assessed via differential scanning calorimetry (DSC), which can provide a helpful understanding of the synthesis and crosslinking behavior of hydrogels [166]. Although it was already mentioned that FTIR can be used to determine phase transitions, DSC is the primary technique used to determine such properties [157]. Properties which can be assessed via DSC analysis include crystallization heat (Hc), melting point (Tm), crystallization temperature (Tc), and melting heat (Hf); water loss and thermal degradation studies can be performed for biopolymers [167].

This technique provides a challenge when assessing certain properties, such as the glass transition, and also, using other techniques to study hydration and study multiple formulations of crosslinked polymers with various structures is advisable to determine the baseline for optimal water content [159].

Thermogravimetric analysis (TGA) is used to study various properties of hydrogels, such as the total mass of water, the mass of dry polymer, the thermal decomposition, the temperature of maximum degradation, and the temperature and rate of dehydration [159]. It is considered an effective and accurate method for measuring bound water in hydrogels, as the derivative thermogravimetric analysis (DTG) curves can be obtained from the reaction rates and used to help identify the free and bound water volumes [168]. However, the technique has low sensitivity and does not measure the phase transitions [159].

### 4.4. X-Ray-Based Techniques

X-ray diffraction (XRD) serves as an excellent mechanism to aid in the investigation of crystalline materials in general, and most studies on hydrogels have focused on this aspect. It is essential to fully understand and characterize the materials by their size, thickness, crystalline perfection, and shape [151]. It can be effectively accompanied by TEM for the determination of the microstructure of a hydrogel [169]. By determining crystallinity, the optical properties of hydrogels can be indirectly assessed through XRD [158].

Despite its utility, XRD has limitations. The sample must be single-phased; the detection limit for mixed materials is about two percent of the sample, and peak overlays could occur for reflections with high angles [170].

### 4.5. Electrochemical Techniques

Miscellaneous electrochemical techniques can be used for the characterization of conductive hydrogels, depending on the property that is the subject of the investigation. Cyclic voltammetry (CV) has been applied to analyze the redox behavior of hydrogels [171] chronoamperometry (CA) can be performed to estimate the effects of natural convection in the hydrogels [172]. Electrical impedance measuring techniques, such as the 4-point probe method, can test the conductive properties of the hydrogels [171]. Investigation of the diffusivity of small molecules and proteins through hydrogels is assessed via electrochemical sensors [173].

Nonetheless, electrochemical characterization techniques are generally used as supplementary methods and not as the main characterization methods.

### 4.6. Rheology

Rheological measurements are crucial for characterizing hydrogels. The rheological parameters, such as viscosity, thixotropy, and viscoelastic behavior, are essential for the understanding of the behaviour of hydrogels [161]. Results obtained from experiments revealed that rotational rheometry can be confidently used to determine viscous and elastic response of hydrogels in the aqueous state, helping to select the right type of gel and amount of concentration for the bio-printing of tissues [174]. There are also methods for the characterization of hydrogels that can simultaneously observe the gelation and polymerization of hydrogels. Rheology–Raman combination is used as a powerful tool for characterizing hydrogels [161].

Despite the successful use of rheology studies, it also has some drawbacks: the method may be destructive, and it is worth noting that the use of different-sized plates and geometries leads to different results despite using the same measuring parameters [175].

## 5. Biological Interactions and Safety Assessment

One of the main reasons for using nanoparticle–hydrogel systems, as discussed above, is the possibility of adjusted, localized, and prolonged delivery of therapeutics, leading to improved biological/medical effects of hydrogels [69,176]. The desired biological effects include antimicrobial, anti-inflammatory and antioxidant activities. Additionally, nanoparticle-hydrogel systems enable drug delivery of more than one therapeutic with different modes of action (for example, delivery of antimicrobial and anti-inflammatory agents at the same time), which is important in multifactorial pathologies such as tumors or microbial infections [42,148].

**Figure 6:**
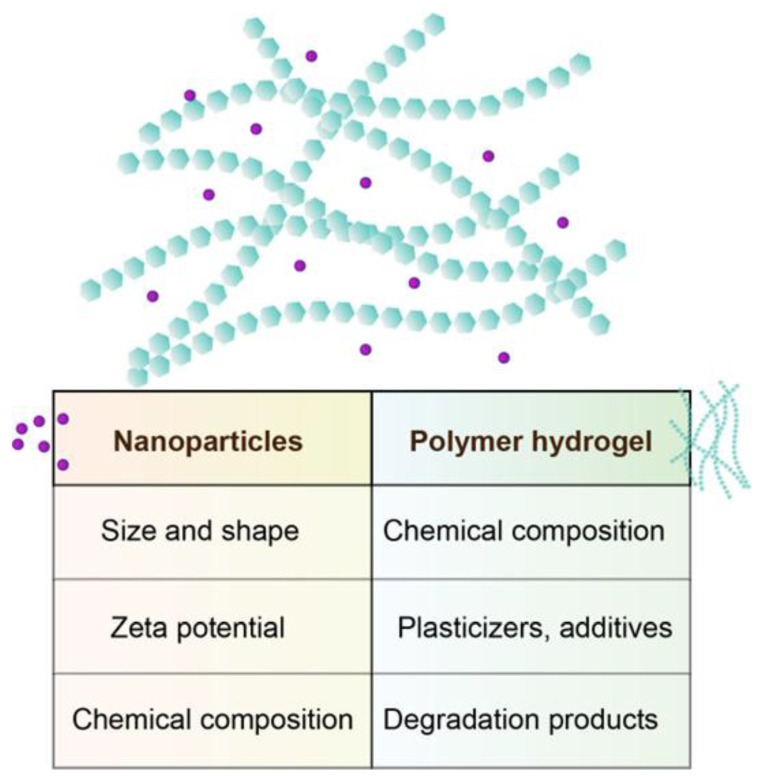
The factors affecting the toxicity of nanoparticle-loaded hydrogels.

However, the limiting property of hydrogels loaded with active agents is their biocompatibility, a compatibility of a material with a biological system, in terms of physiological effects [177]. Usually, the hydrogel matrix is made of polymers that have been proven to be biocompatible and are capable of sustained or stimuli-dependent release of a biologically active agent. Crosslinked hydrogels contain high water content and are generally similar to the structure of the extracellular matrix. This makes the basic polymer structure highly biocompatible and suitable as a platform for cell proliferation [178,179,180] and also offers benefits in wound healing due to the possibility of maintaining adequate moisture levels in the treated wound [148]. There are some differences in the biocompatibility of the polymers, depending on the polymer type and crosslinking method. Generally, hydrogels of synthetic origin and chemically crosslinked have lesser biocompatibility and biological effects than natural, physically crosslinked ones, but the former are sometimes preferred because of better mechanical characteristics [179]. Following contact with the live tissue, hydrogels are developing complex interactions with the surrounding cells. Also, in cases of hydrogel scaffolds seeded with cells before implantation, cells interact with hydrogels, and the whole system reacts with the surrounding tissue, creating an even more complex ambient. Furthermore, in the hydrogels made of several polymers, even if individually they are biocompatible, the possible interactions between them, and also with added agents, should be carefully assessed [177,180]. The nanoparticle part of the system, which is usually the bearer of the desired biological effect, is also potentially hazardous due to toxicity and accumulation [181]. Many NPs, especially the metallic ones, can have toxic effects. Also, nanocarriers for the delivery of therapeutic agents sometimes carry a potentially highly toxic substance, especially in the case of anticancer drugs [69]. It should be emphasised that the observed biological and safety outcomes result from both the hydrogel matrix and the incorporated active agents. While the polymeric hydrogel generally provides a biocompatible and supportive environment, the nanoparticles or loaded therapeutics largely drive specific effects such as antimicrobial, anti-inflammatory, or antioxidative activity. Therefore, the contributions of the hydrogel itself and the active components must be considered distinctly when evaluating cytotoxicity, genotoxicity, immunomodulatory responses, and overall therapeutic performance. Metallic nanoparticles generally present the highest potential risk of toxicity. Their toxic effects depend on several factors, including particle size, surface charge, shape, chemical composition, solubility, and tendency to agglomerate. The main factors affecting the toxicity of nanoparticle-loaded hydrogels have been presented in Figure 6.

### 5.1. Cytotoxicity and Genotoxicity Assays

**Figure 7:**
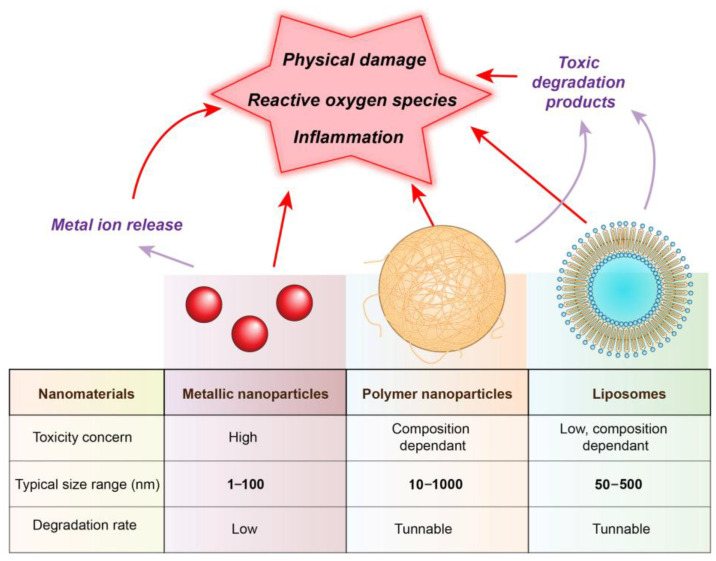
Some of the common toxic effects and characteristics of various nanoparticles.

Due to all of this, the regulation rules and safety requirements regarding biomaterials, and especially nanocomposite biomaterials such as nanoparticle-loaded hydrogels, are very rigorous [181]. It is necessary to prove safety and efficiency before any clinical testing, and the fulfilment of regulatory requirements is assessed by several regulatory bodies, among them the Food and Drug Administration (FDA) or the European Medicines Agency (EMA) [182]. The guidelines for testing of hydrogels and generally medical devices are determined by the International Organisation for Standardisation (ISO) in the form of standards such as ISO 10993 series (Biological Evaluation of Medical Devices) [177,183]. All categories of medical devices must be assessed in terms of the potential cytotoxicity of the material to the cells, and ISO 10993-5 for in vitro cytotoxicity suggests testing the effects of the material extract in the medium, as well as the effects of direct and indirect contact with the cells [183]. Figure 7 gives some of the common toxic effects and characteristics of various nanoparticles.

To measure cytotoxicity, several in vitro assays have been commonly used by researchers. Probably the most popular is the 3-(4,5-dimethylthiazol-2-yl)-2,5-diphenyltetrazolium bromide (MTT) assay, in which the tetrazolium dye, which can be reduced to insoluble purple salts by the living cells, is used as an indicator of viability [181]. Other methods include using resazurin (Alamar blue) viability indicator, measuring the release of lactate dehydrogenase (LDH test), hemolysis assay performed on the blood cells, as well as various fluorescent Live/Dead staining techniques and microscopic techniques [148,181,184,185]. A list of some most common assays used to detect the effects on cell viability is listed in Table 4. Cytotoxicity tests performed on the cells can be done in 2D or in 3D culture, with the latter offering better insight to naturalistic cell response [181]. Also, while assessing the cytotoxicity can be challenging due to nonspecific interactions, attempts have been made to improve the reliability of hydrogel cytotoxicity testing, such as direct cell sampling in MicroDrop format [186]. Besides cytotoxicity, among the frequently used in vitro methods to test for the effects of hydrogels for wound healing is a scratch assay or transwell assay for estimating cell migration [184], or techniques for estimating toxic effects based on cell morphology, markers of apoptosis and necrosis, levels of oxidative stress, effects on mitochondria, proteins, DNA, or calcium levels in the cells [187].

The cells most commonly used as models in such research depend on the biomaterial and the medical device’s future purpose. Commonly used cell lines for studies of wound-healing hydrogels include the human keratinocyte cell line (HaCaT) and the human umbilical vein endothelial cells (HUVEC) [184], while other models, such as mouse L929 fibroblasts [183] or more specialised cell lines, are also frequently used.

Another necessary step is the characterization of in vivo effects of hydrogels in laboratory animals, most often rats and mice, due to their low cost and accessibility [181]. The choice of the animal model depends on the intended purpose. For example, diabetic mice can be used to test the effect of a hydrogel intended for diabetic wound treatment, by measuring the healing time span and the wound surface previously inflicted by a sterile incision [184]. In this type of research, wounds can also be made to be infected or necrotic. By estimating the effect on mice, preliminary data about expected in vivo effects on humans can be obtained. However, in the case of assessing the wound healing potential of hydrogel, more reliable results are complex wounds in porcine models, due to more similar healing mechanisms and skin structure [181].

Besides the acute toxic effects, NPs, especially those of metal and metal oxides, pose risks regarding the possibility of genotoxic effects [192]. Polymers used in hydrogels structure should also be checked in this regard, especially having in mind possible interactions and degradation in the organism. Genotoxicity of hydrogels can be estimated via several assays, such as the bacterial reverse mutation test or chromosome aberration test, for example, used in research by Lee et al. (2022) to confirm the lack of genotoxic effect of the hydrogel based on carboxymethyl chitosan [192], or a comet assay (single cell gel electrophoresis) and micronucleus assay, used to estimate the effects of hydrogels containing iron oxide NPs by Bálintová et al. [193]. Common genotoxicity tests are listed in Table 5.

### 5.2. Inflammation Response

Implantation of medical devices in the organism induces a foreign body immune reaction and subsequent fibrotic scarring. Some materials, especially those known to induce reactive oxygen species (ROS) creation in the cells (such as nanomaterials), can cause a strong inflammation response with consequences leading to the removal of the device and health hazards [108,196,197]. It is therefore important to minimise the risk of inflammation, as well as to adequately estimate the potential of medical devices to cause inflammation. This can be done by activation assays on immune cells, measuring inflammation markers in the cells or in vivo, and by histology staining, biosensors, and other methods [148,184,196,197].

### 5.3. Antioxidative and Anti-Inflammatory Capacity

The beneficial effects of hydrogels used for medicinal purposes are sometimes based on the antioxidative or anti-inflammatory effects to achieve faster healing or suppress inflammation, in addition to anticancer or antibacterial effects [148]. Antioxidants or anti-inflammatory agents in the hydrogel, especially those released by stimuli such as low pH, can significantly improve healing and reduce the risk of chronic inflammation. The NPs in the hydrogel can be the active agents by themselves, but can also carry natural compounds, conventional therapeutics, anti-inflammatory tripeptides, or other agents with positive biological effects [69]. For example, hydrogels loaded with several types of NPs, carrying aspirin, ropivacaine, and lidocaine, lead to 17-fold faster healing of treated wounds [184]. Curcumin-loaded NPs incorporated in the hydrogels showed antigenotoxic effects against the damage induced by aflatoxin B1 in the rat liver [196]. Some metallic NPs in the hydrogels have been shown to reduce mitochondrial membrane potential in the cells, neutrophil activation, and cytokine production, leading to mediation of wound healing [196].

Antioxidative effects of the hydrogels can be tested in vitro without cells, by using assays such as 2,2′-azinobis-(3-ethylbenzothiazoline-6-sulfonate) monocation (ABTS+) radical activity scavenging assay, or other assays based on the colorimetric detection of oxidation of free radicals. The potential as intracellular ROS scavengers can also be tested in live cells by using fluorescence assays such as dichlorofluorescein diacetate (DCFH-DA), which reacts with ROS [197]. Anti-inflammatory effects are usually investigated by measuring the suppressive effect on macrophages and levels of cytokines, most notably NF-κB, TNF-α, IL-6, CD31, PGE2, and other inflammatory mediators [148,184].

### 5.4. Antibacterial Activity

Components such as NPs, plant extracts, or conventional antibiotic agents are added to the porous structure of hydrogels with the aim of controlled release and more efficient antibacterial effects. Certain polymers used in the formation of the gel matrix itself, such as chitosan or alginate, can also have an antibacterial effect [68,198]. Still, the addition of antibacterial NPs to hydrogels may be one of the most efficient methods to ensure antibacterial effect [68]. The NPs in question can be inorganic, metallic/metal oxide particles (which themselves have antimicrobial effect) or can be made as carriers for other antimicrobial agents—antibiotics, antimicrobial peptides, plant extracts. The hydrogel protects the antimicrobial agents from external factors and is also localizing them on the infection site [69,176,198,199]. Hybrid hydrogel–nanoparticle systems can also allow for sustained release and provide other modes of action against pathogens, such as nanoparticle-mediated detoxification of bacterial toxins [176,199]. Among the most common elements used for the synthesis of inorganic NPs are silver, gold, copper, zinc, and their oxides [200]. Furthermore, silver NPs are already one of the commercialized antimicrobial additives to hydrogels [198]. The antibacterial hydrogels can achieve either a bacteriostatic or a bactericidal effect [201]. The mode of action depends on hydrogel components, and sometimes their interactions. In case of NPs, antimicrobial effect may be due to ROS being formed on the NP surface, by mechanical damage to the bacterial cells [198] or other mechanisms. For example, silver ions can damage proteins and inhibit DNA replication in bacterial cells [199]. The testing of antibacterial activity of hydrogels is mostly done by plate colony counting (used to detect the exact viable number of bacteria remaining after the exposure to hydrogel), measuring of zone of inhibition of bacterial growth on agar around the gel sample, measuring of optical density in the treated suspension, and also by scanning electron microscopy and other methods, with zone of inhibition and suspension methods being one of the most commonly used [181,201]. The microbial models used in testing depend on the purpose. For wound healing gels, for example, the most common models are bacterial species *Staphylococcus aureus* and *Pseudomonas aeruginosa* [68,176,181], but also *Escherichia coli, Acinetobacter baumanii, Corynebacterium* spp., and other [181,198]. Furthermore, the effect is often assessed for wound infection-causing fungal species, such as *Candida albicans* or polymicrobial communities [33,68,198].

## 6. Smart and Stimuli-Responsive Hydrogels

### 6.1. Hydrogels with Nanomaterials for Electrophysiology and Electrical Stimulation (EP/ES)

Hydrogels can form soft, water-rich, ionically conductive networks that match tissue mechanics and adhere intimately to skin, nerve, and cardiac surfaces, resulting in reduced electrode–tissue impedance and motion artifacts while improving comfort during long wear. Nanomaterials embedded in these networks (conducting polymers, 2D carbides, graphene, metallic nanostructures) convert the gel into a mixed ion–electron conduit with high stability and processability, enabling both high-fidelity recording of electrophysiological signals (EEG/ECoG/EMG/ECG) and safe stimulation (TENS/FES/neuromodulation/cardiac pacing) [202,203,204]. Table 6 provides a list of specifications that should be met for applications in EP and ES.

#### 6.1.1. Material Platforms

##### Conducting-Polymer Hydrogels

PEDOT:PSS hydrogels provide electronic conductivity with tissue-like modulus; recent work leverages 3D printing to pattern low-impedance, adhesive microelectrodes that record ECG/EMG and survive stretch, sweat, and bending [207,213,214]. Design strategies include nanofibrillar templating and polymer phase-separation to decouple ionic/water pathways from electronic ones, improving charge transport and integrity under large strain [213,215].

##### MXene–Polymer Networks

Ti_3_C_2_T_x_ MXene nanosheets endow gels with high electronic conductivity, broad bandwidth sensing, and photothermal responsiveness while surface terminations (–F, –OH, –O) hydrogen-bond to common polymer hosts (PVA, PAM, alginate), giving adhesion and self-healing. These composites have been used for epidermal EP monitoring (ECG/EMG) and temperature sensing [208]; for ultra-stable gesture-recognition strain sensing that can drive HMI/VR [214]; and for degradable, injectable epidermal sensors [211]. Additional process advances—such as printable MXene inks for transparent e-skin—are pushing toward wafer-scale or roll-to-roll fabrication [216].

##### Graphene/CNT Organohydrogels

Graphene and CNTs create percolating electron networks while the gel phase offers water/ion transport and self-healing. They are effective for EMG capture, robotic control, and e-tattoo-like patches with strong conformability [217,218].

##### Ionic & Adhesive Gels

Ionic double-network gels (e.g., PVA/PEI/CaCl_2_ or zwitterion-rich systems) deliver low skin impedance and strong, reversible adhesion, enabling high-SNR EMG/EEG with less gel drying or irritation [204,205,209]. Beyond these electrically conductive and adhesive systems, MOF–hydrogel composites represent another class of multifunctional ionic/adhesive platforms. By integrating the high porosity and stimuli responsiveness of MOFs with the biocompatibility and injectability of hydrogels, these composites enable high drug loading and localized, sustained, on-demand release. The hydrogel matrix stabilizes MOFs in biological media, minimizing aggregation and burst effects while improving safety and therapeutic performance. Such systems exhibit pH-, ultrasound-, and ROS-responsive release, enhanced tumor penetration, tissue adhesion in cartilage repair, and accelerated wound healing with antibacterial and pro-angiogenic effects [219,220].

##### 4D-Printed Hydrogels

4D-printed hydrogels with programmable, stimuli-responsive shape morphing and integrated conductivity for neural interfaces combine swelling-induced motion, flexibility, and biocompatibility to create self-folding nerve conduits and strain sensors that adapt their geometry and electrical performance under physiological stimuli. Exemplary systems include self-folding conduits for sutureless neurorrhaphy [221] and multifunctional conductive hydrogels incorporating alginate, PVA, and carbon nanofillers for stable neural sensing [222,223].

#### 6.1.2. Recording Performance & Integration

On skin, nanocomposite hydrogels reduce interface impedance and stabilize contact during motion (sweat, shear), lifting SNR for ECG/EMG and enabling reliable, long-wear EEG/ECoG without abrasive preparation [204,205,211]. In the brain, fully hydrated, transparent hydrogel electrodes minimize micromotion and glial response while preserving optics for calcium/voltage imaging [203,206]. Mechanically, matching storage modulus (10^2^–10^3^ Pa for scalp, kPa range for muscle/epicardium) plus tough double networks and nanofillers curbs crack growth under stretch: humectants (glycerol/ionic liquids) and encapsulation slow dehydration for week-long stability [202,224].

### 6.2. Electrical Stimulation with Hydrogels

Conducting polymer and MXene hydrogels are charge-injection interfaces for safe ES that increase charge storage and lower polarization, improving comfort for TENS/FES and reducing threshold for neuromodulation [207,225]. In cardiology, soft hydrogel contacts distribute current uniformly and mitigate fibrotic encapsulation, enabling fully implantable wireless pacing/sensing with stable thresholds in vivo [212]. Closed-loop systems that sense & stimulate (e.g., EMG-triggered FES, neural sensing with adaptive pulses) benefit from hydrogels’ simultaneous ionic/electronic conductivity and low-noise adhesion, allowing compact architectures with rigid-flex PCBs or stretchable leads [210,213]. Furthermore, MXene–hydrogel composites combine exceptional conductivity, mechanical robustness, and responsiveness, enabling advanced sensing, antibacterial, osteogenic, and photothermal functionalities in bioelectronic and regenerative applications [226,227].

### 6.3. Failure Modes and Mitigation Strategies for Nanomaterial-Doped Hydrogel Electrodes in Electrophysiology and Stimulation

For nanomaterial-doped hydrogels used in electrophysiology and electrical stimulation (ES), recurrent failure modes and practical mitigations are as follows:

**Dry-out → impedance drift**—move to organohydrogels or ionic-liquid (IL) gels, tune the backing’s water vapour transmission rate (WVTR), and add humectants; example: transparent organohydrogels have shown stable neural impedance over time (EIS) in practice [203].

**Skin irritation/sensitisation**—use high-purity monomers and remove residuals, add zwitterionic antifouling, and follow ISO 10993 guidance; example: body-temperature–activated ionic adhesive hydrogels demonstrated good tolerance and reusability on skin [205].

**Edge hot-spots during ES**—round/fillet electrode perimeters, add current-spreading meshes, and verify with current-density/thermal maps; example: conformal conducting-hydrogel coatings help smooth fields and stabilise modulation [210].

**Mechanical delamination**—introduce graded-modulus interlayers, apply catechol/silane primers, and microtexture contact surfaces; example: 3D-printed conducting-hydrogel electro-bioadhesive interfaces (EBIs) achieved strong, repeatable adhesion under use [207].

**Electrochemical drift (e.g., Ag/AgCl)**—employ mixed ionic/electronic networks that buffer Cl^−^, minimise DC bias, and prefer biphasic pulses; example: MXene/conducting-polymer hybrids improved interface stability under sensing and stimulation [208].

**Iontophoresis-related transport**—immobilise nanofillers (covalent grafts/shells) to prevent particle/ion leaching under applied current, maintain chloride reservoirs to keep Ag/AgCl at equilibrium and suppress Ag^+^ release and water-electrolysis-driven pH shifts, and enforce charge-balanced waveforms to eliminate net DC, reducing Faradaic reactions and dermal uptake of dopants [205,210].

### 6.4. Translational Roadmap for Hydrogel EP/ES

Converging advances in materials, processing, and system integration are pushing hydrogel-based electrophysiology (EP) and electrical stimulation (ES) from lab prototypes toward dependable clinical and HMI products. First, adhesive ionic hydrogels that resist dehydration and maintain low interfacial impedance enable multi-day recordings with minimal irritation or drift [205,206]. Second, printable hydrogel inks—MXene and conducting polymers such as PEDOT:PSS—now support conformal, transparent electrode arrays with integrated wiring, reducing assembly complexity while preserving softness [207,214,216]. Third, soft implantable hydrogel electrodes have progressed to fully implantable pacers that combine sensing and stimulation with wireless operation, illustrating a path to compliance-matched bioelectronic therapeutics [212].

Near-term priorities include: (i) standardized bench-to-human protocols reporting impedance/SNR under motion, in vivo charge-injection capacity and charge balance for safe ES, and week-scale dehydration metrics to ensure translational relevance [203,206]; (ii) interoperable wireless power/data modules (BLE, NFC, inductive, ultrasound) that mate with hydrogel arrays without compromising softness, enabling long-term, untethered monitoring and therapy [204,212]; and (iii) machine learning-ready datasets pairing raw hydrogel-electrode signals with synchronized motion/physiology labels to advance artifact-robust decoding and closed-loop ES controllers [204,207]. As these elements mature—materials that stay wet and adhere gently, inks that print systems rather than parts, and soft implants that both sense and stimulate—hydrogel EP/ES is poised to transition from niche demonstrations to scalable platforms for neurology, cardiology, rehabilitation, and immersive HMIs.

## 7. Data-Driven Design and Optimisation

Being highly adaptable materials due to their unique physicochemical properties that allow them to mimic diverse cellular and non-cellular processes, hydrogels continue to attract increasing attention. Accordingly, their use in biomedical applications such as drug delivery, tissue engineering, wound healing, and cancer therapy is not surprising [228]. However, as for the other materials, the development of hydrogels relies on experimentation and laboratory manipulation and eventual subsequent material improvement [229]. Thus, to obtain material properties data, the conduction of a large number of experiments is required. Nonetheless, errors in the experimentation lead to errors in the results, finally leading to the unreliability of the gathered data [229]. Therefore, the development of materials, in this case, hydrogels, relies on trial-and-error experimentation, which is time-consuming, inefficient, and costly [229].

Over time, materials informatics has emerged as a powerful interdisciplinary field, driven by the need to accelerate materials research through data-centric approaches. By integrating computational methods, machine learning (ML) and large-scale data analysis, it provides deeper insights into material behaviour in diverse systems and helps the efficient material selection, design as well as discovery of novel materials [230].

### 7.1. The Role of Data Engineering

Data engineering can be described as the design and implementation of systems for collecting, integrating, and transforming massive and diverse datasets, ensuring their efficient administration and availability throughout the data life-cycle. In a close relationship is data quality management focuses on maintaining the data complete, correct, and consistent [231].

Specifically, for hydrogels, data engineering may refer to obtaining large volumes of experimental data, such as polymer types, crosslinkers, and additives for specific applications. ML models can predict hydrogel behaviour, such as swelling ratios, mechanical strength, and degradation rates, based on formulation and environmental conditions [232]. Adequate data engineering pipelines are crucial, since high-quality data makes the foundation of any predictive modelling.

Recently, advances in automation and ML, propelled progress in accelerated hydrogel design by establishing workflows for fast and at-scale HQ data curation through high-throughput experimentation and characterisation, coupled with autonomous experimentation [233,234,235].

### 7.2. Machine Learning (ML) in Hydrogel Modelling

Besides application in adequate data curation, ML algorithms can be applied to uncover complex, often nonlinear, relationships between material variables, providing more flexibility than conventional statistical models [236,237]. As for the hydrogels, ML can be used to enhance various aspects of research, including material design, preparation process optimization, and material characterization. By enabling predictive modeling and efficient data analysis, ML can help accelerate the development of hydrogels with improved functionality and better suitability for specific biomedical and technological applications [238]. It should be noted that ML encompasses three primary strategies. Supervised learning utilizes labeled data to train algorithms for predictive tasks, Unsupervised learning operates on unlabeled data to discover inherent patterns, and Reinforcement Learning (RL) involves algorithms learning through environmental interaction, aiming to maximize rewards over time. To tackle complex tasks considered unsolvable, ML relies on a variety of model families that offer distinct strengths for specific applications [232,238,239,240].

Classical supervised learning models like random forests, support vector machines, logistic regression, partial least squares are used for mapping inputs like polymer type descriptors to hydrogel properties like elasticity, tensile strain, degradation rate, swelling ratio, release kinetics, bioactivity, etc. Data used for developing models is often gathered experimentally, but also via literature screening. Recently, researchers have focused on building efficient workflows for the selection, optimization, and interpretation of models on hydrogel experimental data to get optimal and interpretable models that can help learn key design principles governing properties of hydrogels. For instance, Extreme Gradient Boosting algorithm outperformed other models in the prediction of tensile strain of PVA hydrogels which was subjected further to SHapley Additive exPlanations (SHAP) analysis to reveal critical factors that contribute to the mechanical property [241]. A similar approach was employed in predicting release profiles and kinetic parameters for different formulations of hydrogels [242,243], design of super-adhesive hydrogels [244] or viscosity predictions of developed hybrid hydrogels [245]. Probabilistic models such as the Gaussian Process Regression algorithm emerged as optimal for predicting time-dependent drug release of acetalated dextran nanofibers [246] and rheological properties of methylcellulose [247]. Deep learning models such as artificial neural networks (convolutional, recurrent, graph neural networks, and transformers) are now emerging as tools in hydrogel design [248,249,250,251].

Unsupervised learning techniques are used for pattern (or class) discovery in unlabeled data (clustering, segmentation) and dimensionality reduction using PCA (principal component analysis) for high-dimensional data. The PCA and clustering proved to be very useful tools in data analysis for characterisation methods such as ATR-FTIR, Raman Spectroscopy, or, for instance, Confocal Raman Microspectroscopy [245,252,253,254]. RL methods are also arising as tools for optimisation of hydrogel design [232,255,256]. For example, RL algorithms were integrated into an AI-driven platform that utilizes generative design and multi-objective constrained optimization to generate a novel thiol-containing high-efficiency antimicrobial peptide hydrogels [257].

ML algorithms have great potential in the design of smart hydrogel-based sensors integrated with flexible electronics, as well as in the interpretation of complex output data of such devices, enhancing the responsiveness and sensitivity to environmental stimuli such as temperature, pressure, pH changes, ultrasound, etc. [258,259,260,261,262,263].

### 7.3. Role of Artificial Intelligence (AI) in Personalised Medicine and Hydrogel Design

Personalised medicine applies advanced omics technologies to tailor precise, individualised treatments by identifying disease causes, predicting patient responses, ultimately improving diagnosis, and optimising treatment efficacy while minimising side effects [264,265]. Due to their biocompatibility, flexibility, and water-retaining capacity, hydrogels are widely used in biomedical fields such as ophthalmology, orthopaedics, wound care, and regenerative medicine. When functionalized with nanofibers or peptides, hydrogels enable targeted drug delivery and support tissue regeneration [11]. AI-driven strategies support virtual exploration of formulation spaces, enabling rapid prediction and optimisation of hydrogel properties, which is vital for applications in drug delivery, bioprinting, biosensing, and tissue repair [232,239]. Thus, when coupled with AI, hydrogel-based systems are being transformed from passive carriers into intelligent, responsive agents, capable, for example, of monitoring wounds or tailoring cancer therapies.

Chronic wounds pose a significant clinical challenge due to their complex microenvironment and patient-specific variability. Treatment is often prolonged and costly, placing substantial burdens on healthcare systems worldwide. Factors such as chronic inflammation, bacterial infection, and cellular senescence further delay healing and increase the risk of severe complications like amputation and sepsis [266].

Hydrogels, with their moist and biocompatible structure, are ideal wound dressings. Recently, they have been enhanced by integrating biosensors and AI, enabling personalised, continuous wound monitoring [267]. For instance, thermo-responsive (poly(N-isopropylacrylamide)) (PNIPAm) hydrogels can respond to temperature variances to realise volume phase transitions [268], while conductive hydrogels detect changes via ion migration [269]. Wu et al. developed a deformation-insensitive hydrogel sensor, MXene/clay/PNIPAm (MCP), that maintains accurate temperature detection under mechanical strain [270]. Li et al. designed a multifunctional hydrogel patch combining tannin-grafted gelatin, silver–tannin NPs, polyacrylamide, and poly(N-isopropylacrylamide), integrating temperature sensing with antibacterial and controlled drug release features for intelligent wound care [271].

Oxygen monitoring is essential for assessing wound healing, and hydrogel-based sensors offer high porosity and gas permeability for efficient, real-time detection [272,273]. For instance, thread-based electrochemical sensors with silver hydrogel coatings [274] and self-healing organohydrogel sensors composed of polyacrylamide–chitosan networks with 1,2-propanediol have demonstrated high sensitivity, broad detection range and stability under extreme conditions, enabling accurate oxygen sensing in deep or chronic wounds [275].

Moisture levels in wounds can be effectively monitored and controlled using hydrogel-based sensors, such as polyacrylamide-carrageenan films on polydimethylsiloxane substrates, which adjust their conductivity with humidity changes, or optical hydrogels that detect swelling through laser-based thickness measurements [276,277].

The pH of a wound, often elevated during infection (7.5–9.0), can be assessed using hydrogel-based optical sensors, such as bromocresol purple-functionalized dressings or bacterial nanocellulose with mesoporous silica NPs, both offering visual and high-resolution detection of infection-related pH shifts [278,279]. Additionally, electrochemical sensors with polyaniline-functionalized indium tin oxide electrodes enable real-time, quantitative evaluation of wound pH, with a wireless smartphone readout [267,280].

Glucose-responsive hydrogels, such as those incorporating gelatin methacrylate, glucose oxidase, and pH-sensitive nanogels, enable accurate and rapid detection of glucose levels in chronic wound exudate, supporting diabetic wound management through enzymatic sensing mechanisms [281,282]. Additionally, non-enzymatic fluorescent molecular imprinting technologies offer selective glucose detection via fluorescence changes, further enhancing real-time metabolic assessment in wound care [283].

Uric acid levels, which rise during inflammation and cell death in wound healing, serve as reliable biomarkers of wound severity, particularly in chronic venous ulcers [284,285]. Enzymatic hydrogel-based sensors incorporating urate oxidase, such as polyvinyl alcohol hydrogels [286] or screen-printed biosensors with Prussian blue and NFC connectivity [287], enable highly sensitive, real-time monitoring of uric acid in wound exudate, maintaining performance even under mechanical stress.

Cytokines like IL-6 and TNF-α play a key role in all stages of wound healing, with their fluctuating levels indicating inflammation and healing progression [267]. Hydrogel-based sensors integrated with aptamer-functionalized graphene field-effect transistors enable highly sensitive, real-time detection of cytokines such as TNF-α and IFN-γ, with detection limits as low as 2.75 pM in human fluids [288].

Beyond wound care, AI-driven hydrogels are increasingly being explored in oncology for personalised therapy. For instance, a 3D-bioprinted glioblastoma model using patient-derived glioma stem cells embedded in brain extracellular matrix-based bioinks allows precise modelling of tumour behaviour and drug responses. Similarly, breast cancer models developed using bioprinted co-cultures of tumour and stromal cells embedded in alginate-gelatin hydrogels have effectively mimicked the heterogeneous architecture of tumours and reproduced cell migration dynamics in response to chemical stimuli. Combined with AI, these systems can predict treatment outcomes and guide personalized drug release strategies in real time [289].

Recently, Kaladharan et al. [290] have shown that intelligently designed hydrogel systems, guided by AI, can help precision oncology. Namely, a hydrogel-based micro-chip composed of selectively cross-linked (poly (ethylene glycol) diacrylate) (PEGDA) hydrogels embedded in polydimethylsiloxane channels, enabled parallel screening of 155 drug-dose combinations in HCT-116 colon cancer cells within 2.5 h using only 1 μL of each drug. By integrating an AI-based complex system response (AI-CSR) model, the device rapidly identified optimal synergistic drug combinations, significantly accelerating personalized oncology treatments [290].

The integration of AI with multifunctional hydrogels offers a promising platform for advancing precise and personalized treatment strategies across various medical fields.

## 8. Challenges and Future Perspectives

**Figure 8:**
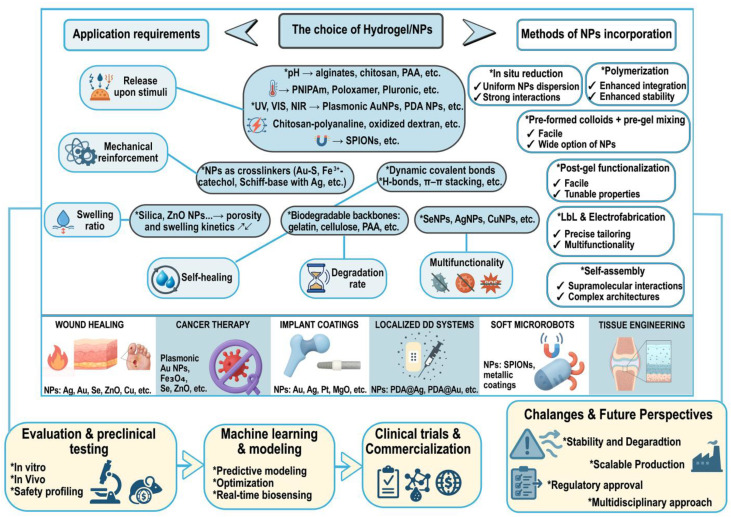
Conceptual framework for the development of nanomaterial-integrated hydrogels. The asterisk (*) indicates emphasis only and has no additional meaning.

While nanomaterial-integrated hydrogels offer remarkable opportunities for advanced drug delivery and different biomedical applications (Figure 8), several challenges continue to limit their clinical translation. A primary concern is the stability of these hybrid systems. Hydrogels are inherently sensitive to environmental conditions such as pH, temperature, and enzymatic activity, which can lead to premature degradation or uncontrolled drug release. Achieving predictable and tunable degradation profiles is equally important, as uncontrolled breakdown not only compromises therapeutic efficacy but may also introduce safety risks through the release of toxic byproducts or non-degradable residues [25].

Another critical challenge lies in the regulatory landscape. The integration of nanomaterials into hydrogel matrices creates complex, multi-component systems that do not fit neatly into existing biomedical product classifications. This complicates safety evaluation, standardisation, and approval processes. Regulatory agencies will need to adapt their frameworks to better address hybrid biomaterials that combine chemical, mechanical, and biological functionalities [180].

From a commercialisation perspective, issues of scalability, reproducibility, and manufacturing cost remain significant bottlenecks. The regulatory challenges for entering the market are especially demanding in the case of biomedical products such as nanoparticle-loaded hydrogels, due to their multicomponent design and intended use. While many polymers used in hydrogel synthesis have already been used for a long time in medicine, the combination with nanoparticles is highly complicated to be properly assessed in terms of regulatory demands. Laboratory-scale synthesis methods—such as hydrothermal or NPs-based strategies—often lack the consistency and economic feasibility required for industrial production. Furthermore, ensuring batch-to-batch uniformity in physicochemical properties is essential for clinical reliability but technically challenging in complex hybrid systems [291]. Looking forward, future progress will rely on a multidisciplinary approach. Advances in computational modelling, machine learning, and real-time biosensing can guide the design of more predictable, robust, and patient-specific hydrogel systems. Long-term studies on biocompatibility, immune responses, and chronic toxicity will be necessary to build confidence in clinical applications. Collaborative efforts between researchers, clinicians, industry stakeholders, and regulatory bodies will play a decisive role in setting standards and accelerating safe translation [232].

Ultimately, overcoming challenges of stability, degradation control, regulatory approval, and scalable production will determine whether nanomaterial-integrated hydrogels can move beyond proof-of-concept to become widely adopted platforms for precision medicine and next-generation drug delivery systems [284,285].

## 9. Conclusions

Nanomaterial-integrated hydrogels are rapidly establishing themselves as next-generation platforms for controlled and sustained drug delivery. Their unique ability to couple the inherent biocompatibility and tunable mechanics of hydrogels with the functional versatility of nanomaterials enables advanced features such as stimuli-responsive release, enhanced bioactivity, and synergistic therapeutic actions. As summarized in this review, progress has been made in synthesis strategies, physicochemical and electrochemical characterization, and evaluation of biological responses including antibacterial, antioxidative, cytotoxic, and genotoxic properties. The integration of data-driven design, particularly through machine learning approaches and wearable biosensors, further expands the potential for personalized and adaptive therapies. Emerging applications, such as functional electrical stimulation and smart responsive delivery systems, highlight the expanding role of these materials in future biomedical practice. Taken together, nanomaterial-integrated hydrogels hold great promise for reshaping drug delivery and therapeutic interventions, provided that challenges of stability, degradation control, regulation, and commercialization are strategically addressed.

## Figures and Tables

**Table 1:** The summary of basic information regarding the preparation of composite hydrogels.

*Method*	*Principle*	*Advantages*	*Limitations*
**In situ *synthesis of*** *NP**s within hydrogels***	Metal precursor ions diffuse into the polymer network and are reduced in situ to form NPs inside the gel.	Uniform particle distribution, strong integration with the matrix, and controlled size.	Requires careful reaction optimization; harsh conditions may damage the gel.
***Mixing pre-synthesized*** *NP**s before gelation***	Stable colloidal dispersions are mixed with the polymer solution, followed by gelation.	Simple and fast; suitable for sensitive biomaterials.	Weaker interaction with the matrix; risk of aggregation.
***Post-gel functionalization***	The hydrogel is formed first and then decorated with NPs through covalent bonding or physical adsorption.	Flexible surface modification; tunable surface properties.	NPs remain mostly at the surface; the procedure can be complex.
***Self-assembly/supramolecular assembly***	NPs and polymers self-assemble into a network via supramolecular interactions.	Enables complex architectures, reversible interactions, and self-healing potential.	Sensitive to environmental changes; requires precise conditions.
***Polymerization in the presence of*** *NP**s***	NPs act as nucleation or anchoring sites during monomer polymerization.	Strong integration into the network; enhanced stability.	Possible toxicity of initiators; requires careful control.
***Layer-by-layer assembly and electrofabrication***	Hydrogels are formed in multiple layers or with multiple networks incorporating NPs.	Advanced functionalities; precise tailoring; improved stability and durability; multifunctionality.	More complex and time-consuming preparation.

**Table 2:** Summary of the examples of hydrogel properties improvement.

Hydrogel (Matrix)	NPs Type	Key Improvements	Application	Reference
**Carboxylated chitosan hydrogel**	Ag NPs (in situ, with berberine)	Stronger antibacterial/anti-biofilm activity; improved stability in infected wounds; mechanical integrity	Wound dressing for drug-resistant bacterial infections	[131]
**Thiolated polyvinyl alcohol/polyethylene glycol diacrylate**	Drug-loaded nanogels (CS/Au nanogels)	Rapid in situ gelation; higher mechanical strength; improved drug retention and localized delivery	Localized breast cancer therapy (injectable in situ gel)	[132]
**Fenugreek polysaccharide hydrogel**	Metal-oxide NPs (MnO_2_, Fe_2_O_3_, CuO formed in situ)	Enhanced thermal stability and mechanical robustness; long-lasting antibacterial and antioxidant activity	Wound healing, antimicrobial coatings, drug delivery	[133]
**pNIPAm/nanogel composite hydrogel**	Linker-modified Au NPs	Higher tensile strength; rapid NIR-triggered self-healing; thermo-responsive swelling; strong skin adhesion	Wearable wound dressings, on-demand therapeutics	[134]
**Gelatin methacryloyl**	CeO_2_ NPs	Improved mechanical stability; antioxidant activity; sustained therapeutic effect; enhanced wound closure	Diabetic wound-healing patch	[135]
**Chitosan-P407-PNIPAm composite hydrogel**	Ag NPs (lipid-complexed)	Enhanced injectability; higher mechanical strength; antibacterial and anti-inflammatory effects; bone regeneration	Periodontitis/alveolar bone regeneration	[136]
**Carrageenan gelatin hydrogel**	Au nanobipyramids (carvacrol loaded)	Photothermal enhancement of chemotherapy; improved local heating and drug release	Cancer therapy (photothermal chemotherapy)	[137]
**Hyaluronic acid-phenylboronic acid hydrogel**	Tea polyphenol-stabilized Ag NPs	Dynamic nano-crosslinking; self-healing; antioxidant and antibacterial activity; controlled release	Diabetic wound healing dressings	[138]
**Locust bean gum/PVA hydrogel**	Ag NPs (green synthesis)	Increased porosity; pH-dependent swelling; improved antibacterial performance; better release control	Antibacterial drug delivery/wound care	[139]
**Laminarin/PVA hydrogel**	Ag NPs (laminarin-reduced)	Improved mechanical stability; accelerated diabetic wound healing; controlled antibacterial release	Diabetic wound healing	[91]
**Gelatin/chitosan hydrogel**	Ag NPs (double-capped with curdlan derivatives)	Enhanced antibacterial efficacy; stronger adhesion; sustained release; improved mechanical robustness	Antibacterial wound dressings	[140]
**Bilayer carrageenan/alginate hydrogel**	Ag NPs (green synthesis)	Effective antibacterial activity; rapid drug delivery; multilayer-controlled release	Rapid wound infection control	[141]
**Hyaluronic acid and quaternized chitosan**	Biomimetic Ag NPs	Injectable and self-healing; antibacterial activity; tailored mechanics	Injectable wound therapy	[142]
**Polyacrylamide hydrogel**	SrO-CoO bimetallic oxide NPs	Improved controlled drug release; stronger mechanics; reduced leaching	Wound healing and controlled release scaffolds	[143]
**Polyacrylamide (PAA) hydrogel crosslinked with MBA (PAA-MBA)**	AgNPs of different shapes: spherical, triangular, rod	Significant increase in storage modulus & Young’s modulus versus NP-free hydrogel; enhanced mechanical strength	Wound dressing, antimicrobial hydrogel materials	[144]
**Methacrylated gelatin hydrogel**	Streamlined-ZnO (non-spherical high-curvature ZnO NPs), PEI/miR-17 complexes	Improved mechanical/rheological properties, MMP-responsive and sustained release of miR-17, enhanced stability, better injectability	Injectable hydrogel for cartilage repair, delivery of miR-17 for osteoarthritis treatment	[145]
**Carboxymethyl tamarind kernel gum (CMTKG)/Poly(sodium acrylate), crosslinked with PEGDA**	ZnO NPs	Improved swelling capacity and thermal stability; increased porosity and gel content; enhanced antibacterial activity; controlled release of ciprofloxacin following Korsmeyer–Peppas (Fickian diffusion) model	Antibacterial hydrogel for controlled drug delivery (ciprofloxacin)	[146]
**PAA@Gelatin**	PDA@Ag (Ag NPs grown on PDA nanospheres)	Enhanced mechanical strength, self-healing, adhesion, reversibility; uniform NP dispersion; higher photothermal conversion efficiency; synergistic antibacterial and analgesic effects	Sustained delivery of lidocaine hydrochloride (LiH); localized long-acting analgesia with NIR-triggered on-demand photothermal pain relief	[147]
**Bacterial cellulose + Gelatin**	Selenium NPs	Enhanced mechanical strength, swelling, flexibility, and biodegradability; slow and sustained release of SeNPs; strong antioxidant, anti-inflammatory, and antibacterial activity (including against multidrug-resistant strains)	Wound dressing for infection prevention and accelerated skin regeneration	[148]

**Table 3:** Comparative overview of nanocomposite hydrogels: influence of nanomaterial type on mechanical properties, release profile, biocompatibility, and application based on responsiveness.

Type of NPs	Mechanical Properties	Release Profile	Biocompatibility	Application	Ref.
**Ag NPs**	↑ Stiffness & toughness; enhanced wet adhesion	Sustained release; prolonged antibacterial effect	No cytotoxicity up to 50 µg/mL; >90% cell viability	Wound dressing; antibacterial coatings	[113,140]
**Au NPs**	Tensile strength ↑ 2×; ~85% self-healing in 2 min under NIR	Precise thermo-responsive release; NIR-triggered delivery	Viability > 85% up to 100 µg/mL; good cytocompatibility	Wound healing; wearable therapeutics; bioinks	[92,111]
**Fe_3_O_4_ NPs**	↑ Elastic modulus; puncture resistance; swelling ↑; strong magnetic responsiveness	Tunable release with external magnetic field (54 → 90%); sustained antibiotic release	Cytocompatible at <200 µg/mL; cell proliferation supported	Ferrogels; cartilage repair; guided drug delivery	[117,118]
**TiO_2_, ZrO_2_ (TMOs)**	↑ Compressive strength, elastic modulus; self-healing	Enhanced release under enzymatic or pH stimuli	Good cytocompatibility; non-toxic at tested levels	Diabetic wound healing; bone/cartilage regeneration	[114,115]
**Se NPs**	Improved flexibility, biodegradability, and toughness	Slow, sustained Se release; antioxidant effect	No significant cytotoxicity; promotes angiogenesis	Wound healing; antimicrobial and anti-inflammatory dressings	[148]
**ZnO NPs**	↑ Porosity and swelling capacity, thermal stability	Ciprofloxacin release fitted Korsmeyer–Peppas model followed by Fickian diffusion	Safe in low–moderate loading; antibacterial action enhanced	Controlled antibiotic release; infection prevention	[146]
**MSNs (SiO_2_)**	↑ Network density; structural stability	Reduced burst release; prolonged IGF-1 release > 144 h	High biocompatibility; no acute toxicity reported	Sustained multidrug delivery; stability of labile drugs	[123]

**Table 4:** Some of the most common methods for estimating effects on cell viability.

Method	Type	Short Description of the Method
**MTT**	Tetrazolium dye indicator 3-(4,5-Dimethylthiazol-2-yl)-2,5-Diphenyltetrazolium Bromide	Tetrazolium dyes react with the live cell enzymes, which convert them to intensely colored formazan crystals. Crystals are then dissolved, and the absorbance is measured [187,188,189].
**MTS**	Tetrazolium dye indicator 3-(4,5-dimethylthiazol-2-yl)-5-(3-carboxymethoxyphenyl)-2-(4-sulfophenyl)-2H-tetrazolium	
**XTT**	Tetrazolium dye indicator 2,3-Bis-(2-Methoxy-4-Nitro-5-Sulfophenyl)-2*H*-Tetrazolium-5-Carboxanilide	
**Cell counting kit-8**	Tetrazolium dye indicator WST-8 (2-(2-methoxy-4-nitrophenyl)-3-(4-nitrophenyl)-5-(2,4-disulfophenyl)-2H-tetrazolium, monosodium salt)	
**Neutral red uptake**	Cationic dye indicator (Dimethyldiaminotoluphenazine)	Viable cells absorb the dye, after which it is located in the lysosomes. After washing out the unabsorbed color, the dye is solubilized, and absorbance is measured [187,189,190].
**Resazurin**	Phenoxazine dye, blue	Redox indicator, cell-permeable. In viable cells is reduced to a fluorescent, pink product, resorufin. Quantification of signal is done by fluorescent spectrophotometry [187,188].
**LDH assays**	Lactate dehydrogenase (LDH) based assays.	LDH is released from dead or damaged cells; therefore, its amount is inversely proportional to the number of healthy cells. Detection can be fluorometric, colorimetric, or bioluminescent [187,189].
**Hemolysis assay**	Measuring the release of hemoglobin	Released hemoglobin is an indicator of the lysis of erythrocytes. It is detected using spectrophotometry [187,191].
**Live/Dead staining techniques**	A combination of fluorescent dyes	The principle of the fluorescent live/dead staining is based on a combination of two different fluorescent dyes, one of which stains live cells, while the other stains dead cells. The detection and quantification are then performed by fluorescent microscopy or cytofluorimetry [184].

**Table 5:** Common genotoxicity tests.

Test Name	Purpose/Description
Bacterial reverse mutation test	Detects the ability of a test substance to induce reverse mutations in specialized bacterial strains that possess easily detectable mutations for certain traits [192].
Chromosome aberration test	Testing of the potential of induction of structural damage in chromosomes in metaphase in mammalian cells (clastogenesis). Uses specialized stains and is based on microscopy. Can be performed on the in vitro or in vivo samples [194].
Micronucleus assay	Testing of damage to chromosomes, structural as well as numerical (aneuploidy). Uses specialized stains and is based on microscopic detection of micronuclei (interphase chromosomes, or chromosome fragments). Can be performed on the in vitro or in vivo samples [194].
Comet assay	Detection of induction of DNA breaks in vitro *or* in vivo. Based on the electrophoresis of DNA fragments and microscopic evaluation [195].

**Table 6:** Specific targets for nanomaterial-doped hydrogels in electrophysiology (EP) & electrical stimulation (ES).

Parameter	Typical Target/Range (Application-Dependent)	Why it Matters (EP/ES)	Design Levers (Nanomaterials & Chemistry)	Verification/Notes	Recent Examples (Last ≤5 yrs)
**Water content**	50–90% (*w*/*w*)	High water lowers skin–electrode impedance, improves comfort & ion transport	Organohydrogels (water–glycerol), zwitterionic/ionic gels; humectants	Gravimetry; mass loss vs. time under use-like RH/temp	[205,206]
**Elastic (Young’s) modulus**	1–100 kPa (skin-like)	Conformality → stable contact, less motion artifact & hot spots	Soft ionogels; catechol/boronate networks; nanofillers to tune	Uniaxial compression/tension; DMA	[202,207]
**Conductivity (ionic +/− electronic)**	10^−4^–10^−2^ S·cm^−1^ (ionic gels) to ≥10^0^ S·cm^−1^ (mixed electronic/ionic)	Low interface Z, stable bi-phasic stimulation, low noise	PEDOT:PSS, MXene, graphene/CNT networks; salt/ILs for ionic pathways	4-point probe/EIS in wet state; temp & frequency sweeps	[203,207,208]
**Skin–electrode impedance (10–100 Hz)**	ECG/EMG: ≤1 kΩ; EEG (hair): typically few–tens kΩ with good SNR	Sets noise floor & stimulation efficiency	Larger area; conductive fillers; chloride buffering; soft fit	EIS on body; report area, pressure, hair/skin prep	[205,209]
**Interfacial impedance @1 kHz**	≲10^2^–10^3^ Ω·cm^2^ (recording); lower preferred for ES	Minimizes polarization, improves charge transfer	Mixed conduction (PEDOT:PSS/MXene), Ag/AgCl buffering	EIS with physiological electrolyte	[207,210]
**Peel adhesion (180°)**	~0.3–1.5 N·cm^−1^	Secure but gentle removal; stable long-wear contact	Catechol/zwitterion/boronate motifs; microtexture	ASTM/ISO peel at controlled rate; cyclic reuse	[205]
**“Reversible” adhesion**	Multi-use on/off with minimal residue & stable Z	Re-positioning; repeated sessions without irritation	Thermo-/moisture-switchable bonds; ionic/zwitterionic gels	Track Z & peel over cycles (10–100×)	[205]
**Dehydration stability**	Minimal drift over 4–72 h	Stable impedance & SNR during wear	Organohydrogels (glycerol/IL), barrier films with tuned WVTR	Mass/impedance drift under wear-like conditions	[203]
**Charge injection capacity & polarization (ES)**	Stable under biphasic pulses; low DC offset	Avoids tissue damage & drift	PEDOT:PSS/MXene networks; chloride reservoirs; smooth edges	Voltage transient analysis; CIC vs. pulse width & density	[207,210]
**Current density uniformity (ES)**	No edge hot-spots (validated maps)	Prevents burns & pain; uniform fields	Filleted geometries; current-spreading meshes/inks	FEA + thermal mapping under load	[210]
**Biocompatibility**	Meets ISO 10993-5/-10/-23	Safety for long-term skin/implants	High-purity monomers; thorough wash; antibacterial without polarization	Cytotox/irritation/sensitization panels	[205,211]
**Application scope**	EP (EEG/ECG/EMG), ES (TENS/EMS), neuromodulation	Versatility across recording + stimulation	Tailor filler type/loading to band & duty	Report per-modality benchmarks	[206,212]

## Data Availability

No new data were created or analyzed in this study. Data sharing is not applicable to this article.
